# Regional Practice Variation and Outcomes in the Standard Versus Accelerated Initiation of Renal Replacement Therapy in Acute Kidney Injury (STARRT-AKI) Trial: A Post Hoc Secondary Analysis

**DOI:** 10.1097/CCE.0000000000001053

**Published:** 2024-02-19

**Authors:** Suvi T. Vaara, Ary Serpa Neto, Rinaldo Bellomo, Neill K. J. Adhikari, Didier Dreyfuss, Martin Gallagher, Stephane Gaudry, Eric Hoste, Michael Joannidis, Ville Pettilä, Amanda Y. Wang, Kianoush Kashani, Ron Wald, Sean M. Bagshaw, Marlies Ostermann, on behalf of the STandard vs. Accelerated initiation of Renal Replacement Therapy in Acute Kidney Injury (STARRT-AKI) Investigators, Sean M Bagshaw, Ron Wald, Neill K.J. Adhikari, Rinaldo Bellomo, Didier Dreyfuss, Bin Du, Martin P. Gallagher, Stéphane Gaudry, Eric A. Hoste, François Lamontagne, Michael Joannidis, Kathleen D. Liu, Daniel F. McAuley, Shay P. McGuinness, Alistair D. Nichol, Marlies Ostermann, Paul M. Palevsky, Haibo Qiu, Ville Pettilä, Antoine G. Schneider, Orla M. Smith, Suvi T. Vaara, Matthew Weir, Rinaldo Bellomo, Glenn M. Eastwood, Leah Peck, Helen Young, Peter Kruger, Gordon Laurie, Emma Saylor, Jason Meyer, Ellen Venz, Krista Wetzig, Craig French, Forbes McGain, John Mulder, Gerard Fennessy, Sathyajith Koottayi, Samantha Bates, Miriam Towns, Rebecca Morgan, Anna Tippett, Andrew Udy, Chris Mason, Elisa Licari, Dashiell Gantner, Jason McClure, Alistair Nichol, Phoebe McCracken, Jasmin Board, Emma Martin, Shirley Vallance, Meredith Young, Chelsey Vladic, Steve McGloughlin, David Gattas, Heidi Buhr, Jennifer Coles, Debra Hutch, James Wun, Louise Cole, Christina Whitehead, Julie Lowrey, Kristy Masters, Rebecca Gresham, Victoria Campbell, David Gutierrez, Jane Brailsford, Loretta Forbes, Lauren Murray, Teena Maguire, Martina NiChonghaile, Neil Orford, Allison Bone, Tania Elderkin, Tania Salerno, Tim Chimunda, Jason Fletcher, Emma Broadfield, Sanjay Porwal, Cameron Knott, Catherine Boschert, Julie Smith, Angus Richardson, Dianne Hill, Graeme Duke, Peter Oziemski, Santiago Cegarra, Peter Chan, Deborah Welsh, Stephanie Hunter, Owen Roodenburg, John Dyett, Nicos Kokotsis, Max Moser, Yang Yang, Laven Padayachee, Joseph Vetro, Himangsu Gangopadhyay, Melissa Kaufman, Angaj Ghosh, Simone Said, Alpesh Patel, Shailesh Bihari, Elisha Matheson, Xia Jin, Tapaswi Shrestha, Kate Schwartz, Martin P. Gallagher, Rosalba Cross, Winston Cheung, Helen Wong, Mark Kol, Asim Shah, Amanda Y. Wang, Zoltan Endre, Celia Bradford, Pierre Janin, Simon Finfer, Naomi Diel, Jonathan Gatward, Naomi Hammond, Anthony Delaney, Frances Bass, Elizabeth Yarad, Hergen Buscher, Claire Reynolds, Nerilee Baker, Michael Joannidis, Romuald Bellmann, Andreas Peer, Julia Hasslacher, Paul Koglberger, Sebastian Klein, Klemens Zotter, Anna Brandtner, Armin Finkenstedt, Adelheid Ditlbacher, Frank Hartig, Dietmar Fries, Mirjam Bachler, Bettina Schenk, Martin Wagner, Thomas Staudinger, Esther Tiller, Peter Schellongowski, Andja Bojic, Eric A. Hoste, Stephanie Bracke, Luc De Crop, Daisy Vermeiren, Fernando Thome, Bianca Chiella, Lucia Fendt, Veronica Antunes, Maisonneuve-Rosemont, Jean-Philippe Lafrance, François Lamontagne, Frédérick D’Aragon, Charles St-Arnaud, Michael Mayette, Élaine Carbonnaeu, Joannie Marchand, Marie-Hélène Masse, Marilène Ladouceur, Alexis F. Turgeon, François Lauzier, David Bellemare, Charles Langis Francoeur, Guillaume LeBlanc, Gabrielle Guilbault, Stéphanie Grenier, Eve Cloutier, Annick Boivin, Charles Delisle-Thibault, Panagiota Giannakouros, Olivier Costerousse, Jean-François Cailhier, François-Martin Carrier, Ali Ghamraoui, Martine Lebrasseur, Fatna Benettaib, Maya Salamé, Dounia Boumahni, Ying Tung Sia, Jean-François Naud, Isabelle Roy, Henry T. Stelfox, Stacey Ruddell, Braden J. Manns, Shelley Duggan, Dominic Carney, Jennifer Barchard, Richard P. Whitlock, Emilie Belley-Cote, Nevena Savija, Alexandra Sabev, Troy Campbell, Thais Creary, Kelson Devereaux, Shira Brodutch, Claudio Rigatto, Bojan Paunovic, Owen Mooney, Anna Glybina, Oksana Harasemiw, Michelle Di Nella, John Harmon, Navdeep Mehta, Louis Lakatos, Nicole Haslam, Francois Lellouche, Mathieu Simon, Ying Tung, Patricia Lizotte, Pierre-Alexandre Bourchard, Bram Rochwerg, Tim Karachi, Tina Millen, John Muscedere, David Maslove, J. Gordon Boyd, Stephanie Sibley, John Drover, Miranda Hunt, Ilinca Georgescu, Randy Wax, Ilan Lenga, Kavita Sridhar, Andrew Steele, Kelly Fusco, Taneera Ghate, Michael Tolibas, Holly Robinson, Matthew A. Weir, Ravi Taneja, Ian M. Ball, Amit Garg, Eileen Campbell, Athena Ovsenek, Sean M. Bagshaw, Sean van Diepen, Nadia Baig, Sheldon Magder, Han Yao, Ahsan Alam, Josie Campisi, Erika MacIntyre, Ella Rokosh, Kimberly Scherr, Stephen Lapinsky, Sangeeta Mehta, Sumesh Shah, Daniel J. Niven, Henry T. Stelfox, Stacey Ruddell, Michael Russell, Kym Jim, Gillian Brown, Kerry Oxtoby, Adam Hall, Luc Benoit, Colleen Sokolowski, Bhanu Prasad, Jag Rao, Shelley Giebel, Demetrios J. Kutsogiannis, Patricia Thompson, Tayne Thompson, Robert Cirone, Kanthi Kavikondala, Mark Soth, France Clarke, Alyson Takaoka, Ron Wald, David Mazer, Karen Burns, Jan Friedrich, David Klein, Gyan Sandhu, Marlene Santos, Imrana Khalid, Jennifer Hodder, Peter Dodek, Najib Ayas, Victoria Alcuaz, Gabriel Suen, Oleksa Rewa, Gurmeet Singh, Sean Norris, Neil Gibson, Castro Arias, Aysha Shami, Celine Pelletier, Neill K.J. Adhikari, Alireza Zahirieh, Andre Amaral, Nicole Marinoff, Navjot Kaur, Adic Perez, Jane Wang, Gregory Haljan, Christopher Condin, Lauralyn McIntyre, Brigette Gomes, Rebecca Porteous, Irene Watpool, Swapnil Hiremath, Edward Clark, Margaret S. Herridge, Felicity Backhouse, M. Elizabeth Wilcox, Karolina Walczak, Vincent Ki, Asheer Sharman, Martin Romano, Sean M. Bagshaw, R.T. Noel Gibney, Adam S. Romanovsky, Oleksa Rewa, Lorena McCoshen, Nadia Baig, Gordon Wood, Daniel Ovakim, Fiona Auld, Gayle Carney, Meili Duan, Xiaojun Ji, Dongchen Guo, Zhili Qi, Jin Lin, Meng Zhang, Lei Dong, Jingfeng Liu, Pei Liu, Deyuan Zhi, Guoqiang Bai, Yu Qiu, Ziqi Yang, Jing Bai, Zhuang Liu, Haizhou Zhuang, Haiman Wang, Jian Li, Mengya Zhao, Xiao Zhou, Xianqing Shi, Baning Ye, Manli Liu, Jing Wu, Yongjian Fu, Dali Long, Yu Pan, Jinlong Wang, Huaxian Mei, Songsong Zhang, Mingxiang Wen, Enyu Yang, Sijie Mu, Jianquan Li, Tingting Hu, Bingyu Qin, Min Li, Cunzhen Wang, Xin Dong, Kaiwu Wang, Haibo Wang, Jianxu Yang, Bin Du, Chuanyao Wang, Dongxin Wang, Nan Li, Zhui Yu, Song Xu, Lan Yao, Guo Hou, Zhou Liu, Liping Lu, Yingtao Lian, Chunting Wang, Jichen Zhang, Ruiqi Ding, Guoqing Qi, Qizhi Wang, Peng Wang, Zhaoli Meng, Man Chen, Xiaobo Hu, Xiandi He, Shibing Zhao, Lele Hang, Rui Li, Suhui Qin, Kun Lu, Shijuan Dun, Cheng Liu, Qi Zhou, Zhenzhen Chen, Jing Mei, Minwei Zhang, Hao Xu, Jincan Lin, Qindong Shi, Lijuan Fu, Qinjing Zeng, Hongye Ma, Jinqi Yan, Lan Gao, Hongjuan Liu, Lei Zhang, Hao Li, Xiaona He, Jingqun Fan, Litao Guo, Yu Liu, Xue Wang, Jingjing Sun, Zhongmin Liu, Juan Yang, Lili Ding, Lulu Sheng, Xingang Liu, Jie Yan, Quihui Wang, Yifeng Wang, Dan Zhao, Shuangping Zhao, Chenghuan Hu, Jing Li, Fuxing Deng, Haibo Qiu, Yi Yang, Min Mo, Chun Pan, Changde Wu, Yingzi Huang, Lili Huang, Airan Liu, Ville Pettilä, Suvi T. Vaara, Anna-Maija Korhonen, Sanna Törnblom, Sari Sutinen, Leena Pettilä, Jonna Heinonen, Eliria Lappi, Taria Suhonen, Sari Karlsson, Sanna Hoppu, Ville Jalkanen, Anne Kuitunen, Markus Levoranta, Jaakko Långsjö, Sanna Ristimäki, Kaisa Malila, Anna Wootten, Simo Varila, Mikko J Järvisalo, Outi Inkinen, Satu Kentala, Keijo Leivo, Paivi Haltia, Didier Dreyfuss, Jean-Damien Ricard, Jonathan Messika, Abirami Tiagarajah, Malo Emery, Aline Dechanet, Coralie Gernez, Damien Roux, Laurent Martin-Lefevre, Maud Fiancette, Isabelle Vinatier, Jean Claude Lacherade, Gwenhaël Colin, Christine Lebert, Marie-Ange Azais, Aihem Yehia, Caroline Pouplet, Matthieu Henry- Lagarrigue, Amélie Seguin, Laura Crosby, Julien Maizel, Dimitri Titeca-Beauport, Alain Combes, Ania Nieszkowska, Paul Masi, Alexandre Demoule, Julien Mayaux, Martin Dres, Elise Morawiec, Maxens Decalvele, Suela Demiri, Morgane Faure, Clémence Marios, Maxime Mallet, Marie Amélie Ordon, Laura Morizot, Marie Cantien, François Pousset, Stéphane Gaudry, Florent Poirson, Yves Cohen, Laurent Argaud, Martin Cour, Laurent Bitker, Marie Simon, Romain Hernu, Thomas Baudry, Sylvie De La Salle, Adrien Robine, Nicholas Sedillot, Xavier Tchenio, Camille Bouisse, Sylvie Roux, Davide Barbar, Rémi Trusson, Fabienne Tamion, Steven Grangé, Dorothée Carpentier, Guillaume Chevrel, Luis Ensenyat-Martin, Sophie Marque, Jean-Pierre Quenot, Pascal Andreu, Auguste Dargent, Audrey Large, Nicolas Chudeau, Mickael Landais, Benoit Derrien, Jean Christophe Callahan, Christophe Guitton, Charlène Le Moal, Alain Robert, Karim Asehnoune, Raphaël Cinotti, Nicolas Grillot, Dominique Demeure, Christophe Vinsonneau, Imen Rahmani, Mehdi Marzouk, Thibault Dekeyser, Caroline Sejourne, Mélanie Verlay, Fabienne Thevenin, Lucie Delecolle, Lens Didier Thevenin, Bertrand Souweine, Elisabeth Coupez, Mireille Adda, Jean-Pierre Eraldi, Antoine Marchalot, Nicolas De Prost, Armand Mekontso Dessap, Keyvan Razazi, Ferhat Meziani, Julie Boisrame-Helms, Raphael Clere-Jehl, Xavier Delabranche, Christine Kummerlen, Hamid Merdji, Alexandra Monnier, Yannick Rabouel, Hassene Rahmani, Hayat Allam, Samir Chenaf, Vincenta Franja, Bertrand Pons, Michel Carles, Frédéric Martino, Régine Richard, Benjamin Zuber, Guillaume Lacave, Karim Lakhal, Bertrand Rozec, Hoa Dang Van, Éric Boulet, René Dubos, Fouad Fadel, Cedric Cleophax, Nicolas Dufour, Caroline Grant, Marie Thuong, Jean Reignier, Emmanuel Canet, Laurent Nicolet, Thierry Boulain, Mai-Anh Nay, Dalila Benzekri, François Barbier, Anne Bretagnol, Toufik Kamel, Armelle Mathonnet, Grégoire Muller, Marie Skarzynski, Julie Rossi, Amandine Pradet, Sandra Dos Santos, Aurore Guery, Lucie Muller, Luis Felix, Julien Bohé, Guillaume Thiéry, Nadia Aissaoui, Damien Vimpere, Morgane Commeureuc, Jean-Luc Diehl, Emmanuel Guerot, Orfeas Liangos, Monika Wittig, Alexander Zarbock, Mira Küllmar, Thomas van Waegeningh, Nadine Rosenow, Alistair D. Nichol, Kathy Brickell, Peter Doran, Patrick T. Murray, Giovanni Landoni, Rosalba Lembo, Alberto Zangrillo, Giacomo Monti, Margherita Tozzi, Matteo Marzaroli, Gaetano Lombardi, Gianluca Paternoster, Michelangelo Vitiello, Shay McGuinness, Rachael Parke, Magdalena Butler, Eileen Gilder, Keri-Anne Cowdrey, Samantha Wallace, Jane Hallion, Melissa Woolett, Philippa Neal, Karina Duffy, Stephanie Long, Colin McArthur, Catherine Simmonds, Yan Chen, Rachael McConnochie, Lynette Newby, David Knight, Seton Henderson, Jan Mehrtens, Stacey Morgan, Anna Morris, Kymbalee Vander Hayden, Tara Burke, Matthew Bailey, Ross Freebairn, Lesley Chadwick, Penelope Park, Christine Rolls, Liz Thomas, Ulrike Buehner, Erin Williams, Jonathan Albrett, Simon Kirkham, Carolyn Jackson, Troy Browne, Jennifer Goodson, David Jackson, James Houghton, Owen Callender, Vicki Higson, Owen Keet, Clive Dominy, Paul Young, Anna Hunt, Harriet Judd, Cassie Lawrence, Shaanti Olatunji, Yvonne Robertson, Charlotte Latimer-Bell, Deborah Hendry, Agnes Mckay-Vucago, Nina Beehre, Eden Lesona, Leanlove Navarra, Chelsea Robinson, Ryan Jang, Andrea Junge, Bridget Lambert, Antoine G. Schneider, Michel Thibault, Philippe Eckert, Sébastien Kissling, Erietta Polychronopoulos, Elettra Poli, Marco Altarelli, Madeleine Schnorf, Samia Abed Mallaird, Claudia Heidegger, Aurelie Perret, Philippe Montillier, Frederic Sangla, Seigenthaller Neils, Aude De Watteville, Mandeep-Kaur Phull, Aparna George, Nauman Hussain, Tatiana Pogreban, Steve Lobaz, Alison Daniels, Mishell Cunningham, Deborah Kerr, Alice Nicholson, Pradeep Shanmugasundaram, Judith Abrams, Katarina Manso, Geraldine Hambrook, Elizabeth McKerrow, Juvy Salva, Stephen Foulkes, Matthew Wise, Matt Morgan, Jenny Brooks, Jade Cole, Tracy Michelle Davies, Helen Hill, Emma Thomas, Marcela Vizcaychipi, Behrad Baharlo, Jaime Carungcong, Patricia Costa, Laura Martins, Ritoo Kapoor, Tracy Hazelton, Angela Moon, Janine Musselwhite, Ben Shelley, Philip McCall, Marlies Ostermann, Gill Arbane, Aneta Bociek, Martina Marotti, Rosario Lim, Sara Campos, Neus Grau Novellas, Armando Cennamo, Andrew Slack, Duncan Wyncoll, Luigi Camporota, Simon Sparkes, Rosalinde Tilley, Austin Rattray, Gayle Moreland, Jane Duffy, Elizabeth McGonigal, Philip Hopkins, Clare Finney, John Smith, Harriet Noble, Hayley Watson, Claire-Louise Harris, Emma Clarey, Eleanor Corcoran, James Beck, Clare Howcroft, Nora Youngs, Elizabeth Wilby, Bethan Ogg, Adam Wolverson, Sandra Lee, Susie Butler, Maryanne Okubanjo, Julia Hindle, Ingeborg Welters, Karen Williams, Emily Johnson, Julie Patrick-Heselton, David Shaw, Victoria Waugh, Richard Stewart, Esther Mwaura, Lynn Wren, Louise Mew, Sara-Beth Sutherland, Jane Adderley, Jim Ruddy, Margaret Harkins, Callum Kaye, Teresa Scott, Wendy Mitchell, Felicity Anderson, Fiona Willox, Vijay Jagannathan, Michele Clark, Sarah Purv, Andrew Sharman, Megan Meredith, Lucy Ryan, Louise Conner, Cecilia Peters, Dan Harvey, Ashraf Roshdy, Amy Collins, Malcolm Sim, Steven Henderson, Nigel Chee, Sally Pitts, Katie Bowman, Maria Dilawershah, Luke Vamplew, Elizabeth Howe, Paula Rogers, Clara Hernandez, Clara Prendergast, Jane Benton, Alex Rosenberg, Lui G. Forni, Alice Grant, Paula Carvelli, Ajay Raithatha, Sarah Bird, Max Richardson, Matthew Needham, Claire Hirst, Jonathan Ball, Susannah Leaver, Luisa Howlett, Carlos Castro Delgado, Sarah Farnell-Ward, Helen Farrah, Geraldine Gray, Gipsy Joseph, Francesca Robinson, Ascanio Tridente, Clare Harrop, Karen Shuker, Derek McLaughlan, Judith Ramsey, Sharon Meehan, Bernd Oliver Rose, Rosie Reece-Anthony, Babita Gurung, Tony Whitehouse, Catherine Snelson, Tonny Veenith, Andy Johnston, Lauren Cooper, Ron Carrera, Karen Ellis, Emma Fellows, Samanth Harkett, Colin Bergin, Elaine Spruce, Liesl Despy, Stephanie Goundry, Natalie Dooley, Tracy Mason, Amy Clark, Gemma Dignam, Geraldine Ward, Ben Attwood, Penny Parsons, Sophie Mason, Michael Margarson, Jenny Lord, Philip McGlone, Luke E. Hodgson, Indra Chadbourn, Raquel Gomez, Jordi Margalef, Rinus Pretorius, Alexandra Hamshere, Joseph Carter, Hazel Cahill, Lia Grainger, Kate Howard, Greg Forshaw, Zoe Guy, Kianoush B. Kashani, Robert C. Albright Jr., Amy Amsbaugh, Anita Stoltenberg, Alexander S. Niven, Matthew Lynch, AnnMarie O’Mara, Syed Naeem, Sairah Sharif, Joyce McKenney Goulart, Matthew Lynch, AnnMarie O’Mara, Syed Naeem, Sairah Sharif, Joyce McKenney Goulart, Ashita Tolwani, Claretha Lyas, Laura Latta, Azra Bihorac, Haleh Hashemighouchani, Philip Efron, Matthew Ruppert, Julie Cupka, Sean Kiley, Joshua Carson, Peggy White, George Omalay, Sherry Brown, Laura Velez, Alina Marceron, Javier A. Neyra, Juan Carlos Aycinena, Madona Elias, Victor M. Ortiz-Soriano, Caroline Hauschild, Robert Dorfman

**Affiliations:** 1 Department of Perioperative and Intensive Care, University of Helsinki and Helsinki University Hospital, Helsinki, Finland.; 2 Australian and New Zealand Intensive Care Research Centre (ANZIC-RC), School of Public Health and Preventive Medicine, Monash University, Melbourne, VIC, Australia.; 3 Department of Critical Care, Melbourne Medical School, University of Melbourne, Austin Hospital, Melbourne, VIC, Australia.; 4 Department of Intensive Care, Austin Hospital, Melbourne, VIC, Australia.; 5 Department of Critical Care Medicine, Hospital Israelita Albert Einstein, Sao Paulo, Brazil.; 6 Data Analytics Research & Evaluation, Austin Hospital, Melbourne, VIC, Australia.; 7 Department of Intensive Care, Royal Melbourne Hospital, Melbourne, VIC, Australia.; 8 Department of Critical Care Medicine, Sunnybrook Health Sciences Centre, Toronto, ON, Canada.; 9 Institute of Health Policy, Management and Evaluation, Dalla Lana School of Public Health, University of Toronto, Toronto, ON, Canada.; 10 Interdepartmental Division of Critical Care Medicine, University of Toronto, Toronto, ON, Canada.; 11 French National Institute of Health and Medical Research (INSERM), UMR_S1155, CORAKID, Hôpital Tenon, Sorbonne Université, Paris, France.; 12 Service de Médecine Intensive Réanimation, Hôpital Louis Mourier, Assistance Publique, Université de Paris-Cité, Paris, France.; 13 South Western Sydney Clinical Campus, Faculty of Medicine & Health, University of New South Wales, New South Wales, NSW, Australia.; 14 The George Institute for Global Health, University of New South Wales, New South Wales, Australia.; 15 AP-HP, Hôpital Avicenne, Service de Réanimation Médico-Chirurgicale, UFR SMBH, Université Sorbonne Paris Nord, Bobigny, France.; 16 Intensive Care Unit, Department of Internal Medicine and Pediatrics, Ghent University Hospital, Ghent University, Ghent, Belgium.; 17 Division of Intensive Care and Emergency Medicine, Department of Internal Medicine, Medical University Innsbruck, Innsbruck, Austria.; 18 The Faculty of Medicine and Medical Sciences, Macquarie University, Sydney, NSW, Australia.; 19 Division of Nephrology and Hypertension, Division of Pulmonary and Critical Care Medicine, Department of Medicine, Mayo Clinic College of Medicine, Rochester, MN.; 20 Medicine Program and Li Ka Shing Knowledge Institute, St. Michael’s Hospital, Unity Health Toronto, Toronto, ON, Canada.; 21 Division of Nephrology, St. Michael’s Hospital and the University of Toronto and the Li Ka Shing Knowledge Institute of St. Michael’s Hospital, Toronto, ON, Canada.; 22 Department of Critical Care Medicine, Faculty of Medicine and Dentistry, University of Alberta and Alberta Health Services, Edmonton, AB, Canada.; 23 Department of Critical Care Medicine, King’s College London, Guy’s & St Thomas’ Hospital, London, United Kingdom.

**Keywords:** acute kidney injury, continuous renal replacement therapy, fluid balance, intermittent hemodialysis, outcomes, randomized controlled trial

## Abstract

**OBJECTIVES:**

Among patients with severe acute kidney injury (AKI) admitted to the ICU in high-income countries, regional practice variations for fluid balance (FB) management, timing, and choice of renal replacement therapy (RRT) modality may be significant.

**DESIGN:**

Secondary post hoc analysis of the STandard vs. Accelerated initiation of Renal Replacement Therapy in Acute Kidney Injury (STARRT-AKI) trial (ClinicalTrials.gov number NCT02568722).

**SETTING:**

One hundred-fifty-three ICUs in 13 countries.

**PATIENTS:**

Altogether 2693 critically ill patients with AKI, of whom 994 were North American, 1143 European, and 556 from Australia and New Zealand (ANZ).

**INTERVENTIONS:**

None.

**MEASUREMENTS AND MAIN RESULTS:**

Total mean FB to a maximum of 14 days was +7199 mL in North America, +5641 mL in Europe, and +2211 mL in ANZ (*p* < 0.001). The median time to RRT initiation among patients allocated to the standard strategy was longest in Europe compared with North America and ANZ (*p* < 0.001; *p* < 0.001). Continuous RRT was the initial RRT modality in 60.8% of patients in North America and 56.8% of patients in Europe, compared with 96.4% of patients in ANZ (*p* < 0.001). After adjustment for predefined baseline characteristics, compared with North American and European patients, those in ANZ were more likely to survive to ICU (*p* < 0.001) and hospital discharge (*p* < 0.001) and to 90 days (for ANZ vs. Europe: risk difference [RD], –11.3%; 95% CI, –17.7% to –4.8%; *p* < 0.001 and for ANZ vs. North America: RD, –10.3%; 95% CI, –17.5% to –3.1%; *p* = 0.007).

**CONCLUSIONS:**

Among STARRT-AKI trial centers, significant regional practice variation exists regarding FB, timing of initiation of RRT, and initial use of continuous RRT. After adjustment, such practice variation was associated with lower ICU and hospital stay and 90-day mortality among ANZ patients compared with other regions.

**KEY POINTS**

**Question:** Are there variations in practice in the management of critically ill patients with acute kidney injury across major geographical regions (North America, Europe, and Australia and New Zealand [ANZ])?

**Findings:** Among 2693 patients enrolled in an international randomized controlled trial, we found significant differences in baseline patient characteristics, fluid balance, timing, and choice of first renal replacement therapy modality according to major geographic regions. After adjusting for differences in predefined baseline characteristics, ANZ patients had significantly lower 90-day mortality than those in North America and Europe.

**Meaning:** Significant regional variations in practice were found that may be associated with differences in patient outcomes.

The management of critically ill patients with severe acute kidney injury (AKI) is complex. Furthermore, some key aspects of treatment, such as the management of fluid balance (FB), timing, and choice of initial renal replacement therapy (RRT), remain controversial and practice is variable (1–9). Prior data have shown regional variations in the characteristics of patients with AKI who are supported in ICU settings (10–12). Furthermore, clinical trials investigating the intensity of RRT have demonstrated differences in the practices regarding the use of intermittent RRT across regions (13, 14). Additionally, observational studies have reported significant heterogeneity in RRT practice patterns within North America (15, 16). Regarding FB management using ultrafiltration, an international survey has revealed significant practice variation (4), also regionally in North America (17) and Europe (18). Finally, surveys in the United Kingdom (19) and Australia and New Zealand (ANZ) (20) have demonstrated varying practices within nations.

The STandard vs. Accelerated initiation of Renal Replacement Therapy in Acute Kidney Injury (STARRT-AKI) trial was an international multicenter randomized controlled trial conducted in 168 centers across 15 countries. It compared two strategies of RRT initiation in patients with severe AKI who were eligible for RRT initiation but had no urgent indications for RRT initiation (7, 21, 22).

In this post hoc secondary analysis of the STARRT-AKI trial, we aimed to test the hypothesis that, among patients treated in high-income countries, there would be significant practice variation according to geographical regions in relation to: 1) FB management; 2) timing of RRT initiation among patients allocated to the standard RRT strategy; and 3) choice of initial RRT modality. We further hypothesized that such variation would be associated with differences in patient outcomes.

## MATERIALS AND METHODS

### Patients

**Figure 1.**
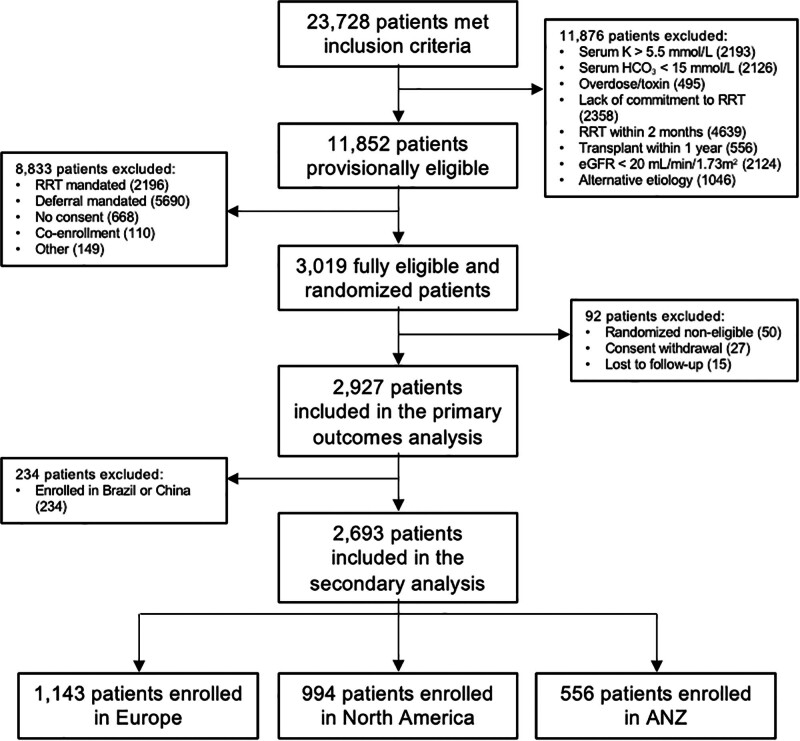
Study flow chart. ANZ = Australia and New Zealand, eGFR = estimated glomerular filtration rate, Hco_3_ = bicarbonate, RRT = renal replacement therapy.

STARRT-AKI enrolled 3019 critically ill adults with severe AKI (stages 2–3 using the Kidney Disease Improving Global Outcomes classification) from 168 centers in 15 countries and five continents (7) (**Fig. 1**). The trial protocol, statistical analysis plan, and the details of the main trial findings have been published (7, 21, 22). STARRT-AKI was registered at ClinicalTrials.gov (ClinicalTrials.gov Identifier: NCT02568722) and was approved by the health research ethics boards at the University of Alberta (File No. Pro00060023), Unity Health Toronto (Clinical Trials Ontario Project Identifier: 0761) and research ethics boards at all participating sites (**eTable 1**, http://links.lww.com/CCX/B310) (7). Informed consent was obtained from participants, substitute decision-makers and/or deferred or waived, as per local health research ethics board approval. The trial was conducted according to the Declaration of Helsinki and its later amendments.

### Exposures

This post hoc secondary analysis aimed to evaluate regional differences in FB management, timing of RRT initiation in the standard arm, and the choice of the initial modality of RRT (Data Creation Plan available at: https://www.ualberta.ca/critical-care/media-library/documents/dcp_starrt-aki-regional-practice-variation-effect-statusv2_july-18-2022.pdf).

We pre-designated geographic regions of high-income regions: North America (Canada and United States); Europe (Austria, Belgium, Finland, France, Germany, Ireland, Italy, Switzerland, and United Kingdom), and ANZ for comparison. Patients from China (*n* = 255) and Brazil (*n* = 8) were excluded because the number of centers and patients treated in these countries was insufficient for meaningful comparisons and because both are upper middle-income countries.

Among patients with available data, we evaluated cumulative FB from the time of ICU admission over their entire ICU stay up to day 14 (7), the timing of initiation of RRT among those allocated to the standard strategy, and the choice of initial RRT modality according to geographic region. Patients discharged from the ICU before day 14 or who died were censored at the time of discharge or death.

### Outcomes

The primary outcome was all-cause 90-day mortality. Secondary outcomes for this analysis were ICU and hospital mortality, ICU-free days at day 28, hospital-free days at day 90, and mechanical ventilation-free days at day 28. A “free day” was considered a 24-hour period in which less than 2 hours were spent in the ICU, in hospital or receiving invasive mechanical ventilation, respectively.

We also obtained kidney-specific secondary outcomes, including RRT dependence at day 90, the composite of mortality and persistent use of RRT at 90 days, and RRT-free days at day 90.

### Sensitivity Analyses

As patient management may be different in medical and surgical patients, especially regarding FB, we performed several sensitivity analyses: 1) among medical patients only, 2) excluding cardiac surgical patients, and 3) excluding all elective surgical patients. Additionally, we performed separate comparisons according to the allocated RRT initiation strategy. Finally, we analyzed geographic regions by dividing Europe into French and non-French European centers. This subdivision was based on the premises that these jurisdictions have differing healthcare systems, have traditionally shown differences in approach to RRT, and had sufficient sample size to enable meaningful comparison (3, 8).

### Statistical Analyses

We applied the intention-to-treat approach as used in the primary trial analysis. Continuous variables are reported as median (quartile 25th–quartile 75th) and compared using the Wilcoxon rank-sum or Kruskal-Wallis test. Categorical variables are reported using absolute numbers (percentage) and compared using Fisher exact test.

Binary outcomes were compared between groups using risk difference (and 95% CI) calculated with a generalized linear model with a binomial distribution and identity link. We used the risk difference for description of varying practices and associated outcomes to minimize attribution of judgment when directly comparing geographic regions. Continuous outcomes were compared between groups using median difference (and 95% CI) calculated with a median regression using an interior point algorithm with CI and *p* values calculated after bootstrapping with 1000 samples. Bootstrap was used to construct robust ses as described elsewhere (23). In addition to univariable models, multivariable models were performed after adjustment for prespecified covariates, including age, sex, Simplified Acute Physiology Score (SAPS) II score, type of admission (surgical vs. medical), and presence of sepsis. These covariates were chosen because they aligned with the main STARRT-AKI statistical analysis plan (7, 21, 22). The study site was also included as a random effect. The supplement provides **Additional Methods** (http://links.lww.com/CCX/B310) about non-normally distributed data and about variable selection for sensitivity analyses. Finally, 90-day survival was reported in Kaplan-Meier curves and compared between groups using the log-rank test.

All analyses were performed using R (Version 4; R Project for Statistical Computing, Vienna, Austria. Available at: https://www.r-project.org/). Given multiple comparisons, the significance level was adjusted with the Bonferroni method. A *p* value of less than 0.01 was considered statistically significant.

## RESULTS

### Patient Characteristics According to Geographic Region

We included 2927 patients with complete data after randomization in this analysis. After exclusion of patients enrolled in China and Brazil, we analyzed 994 patients enrolled in North America, 1143 in Europe, and 556 in ANZ (**Table 1** and Fig. 1).

**TABLE 1.** Baseline Characteristics

Characteristic	North America (*n* = 994)	Europe (*n* = 1143)	Australia and New Zealand (*n* = 556)	*p*
Age, yr	65.4 (56.7–73.5)	68.4 (58.9–76.0)	66.7 (56.6–75.0)	< 0.001
Male gender	672 (67.6)	795 (69.6)	367 (66.0)	0.293
Weight, kg	89.0 (73.6–108.0)	82.0 (70.5–95.0)	87.0 (73.6–102.4)	< 0.001
Simplified Acute Physiology Score II	62.0 (50.0–74.0)	55.0 (44.0–69.0)	61.0 (47.0–74.2)	< 0.001
Randomization group				0.997
Accelerated arm	497 (50.0)	572 (50.0)	277 (49.8)	
Standard arm	497 (50.0)	571 (50.0)	279 (50.2)	
Hours between randomization and renal replacement therapy	7.5 (4.2–26.0)	12.1 (8.7–32.3)	22.6 (19.3–36.8)	< 0.001
Accelerated arm	5.1 (3.2–7.0)	9.3 (7.9–11.8)	6.3 (4.2–9.0)	< 0.001
Standard arm	28.9 (21.0–58.9)	52.5 (29.8–84.0)	40.9 (31.1–63.3)	< 0.001
Initial modality				< 0.001
Continuous renal replacement therapy	481 (60.8)	492 (56.8)	422 (95.7)	
Intermittent hemodialysis	238 (30.1)	361 (41.7)	3 (0.7)	
Sustained low-efficiency daily dialysis	72 (9.1)	13 (1.5)	16 (3.6)	
Type of admission				< 0.001
Medical	686 (69.0)	854 (74.7)	267 (48.0)	
Scheduled surgery	114 (11.5)	108 (9.4)	116 (20.9)	
Unscheduled surgery	194 (19.5)	181 (15.8)	173 (31.1)	
Pre-randomization clinical frailty score	3.0 (2.0–4.0)	2.0 (0.0–4.0)	3.0 (1.0–4.0)	< 0.001
Pre-randomization signs				
Respiratory rate, breaths/min	24.0 (18.0–29.0)	23.0 (18.0–29.0)	20.0 (16.0–24.0)	< 0.001
Positive end-expiratory pressure, cm H_2_O	10.0 (8.0–12.0)	8.0 (5.0–10.0)	8.0 (6.0–10.0)	< 0.001
Cumulative fluid balance, mL	4267 (1777–8827)	2400 (750–4650)	2103 (626–4405)	< 0.001
Pre-randomization blood tests				
pH	7.32 (7.26–7.38)	7.34 (7.27–7.39)	7.33 (7.26–7.39)	0.019
Creatinine, µmol/L	295.0 (226.0–399.0)	265.6 (202.0–359.0)	260.0 (208.5–356.5)	< 0.001
Hemoglobin, g/L	90.0 (79.0–106.0)	102.0 (86.0–118.0)	96.0 (83.0–116.0)	< 0.001
Platelets, ×10^9^/L	140.0 (78.0–217.0)	166.0 (96.0–250.0)	148.0 (90.8–218.2)	< 0.001
Pre-randomization support				
Mechanical ventilation or continuous positive airway pressure	807 (88.9)	864 (75.6)	440 (79.1)	< 0.001
Norepinephrine use	645 (64.9)	726 (63.5)	410 (73.7)	< 0.001
Norepinephrine dose, µg/kg/min	0.2 (0.1–0.3)	0.3 (0.1–0.7)	0.2 (0.1–0.3)	< 0.001
Diuretic use	340 (34.2)	289 (25.3)	233 (41.9)	< 0.001

Data are median (quartile 25th–quartile 75th) or *n* (%).

There were multiple regional differences in baseline patient characteristics, risk factors for AKI, and pre-randomization management (Table 1; and **eTable 2**, http://links.lww.com/CCX/B310). For example, European patients were the oldest and most likely to be admitted with a medical condition. Patients from ANZ were more likely to be admitted after surgery and with a primary cardiovascular admission diagnosis. Sepsis was the primary ICU admission diagnosis in 25.6% of patients from North America and Europe. Patients from ANZ had the highest exposure to cardiopulmonary bypass and IV contrast media (eTable 2, http://links.lww.com/CCX/B310). North American patients had the largest proportion receiving mechanical ventilation, whereas ANZ had the largest proportion receiving norepinephrine and diuretics (Table 1).

### Fluid Balance According to Geographic Region

**Figure 2.**
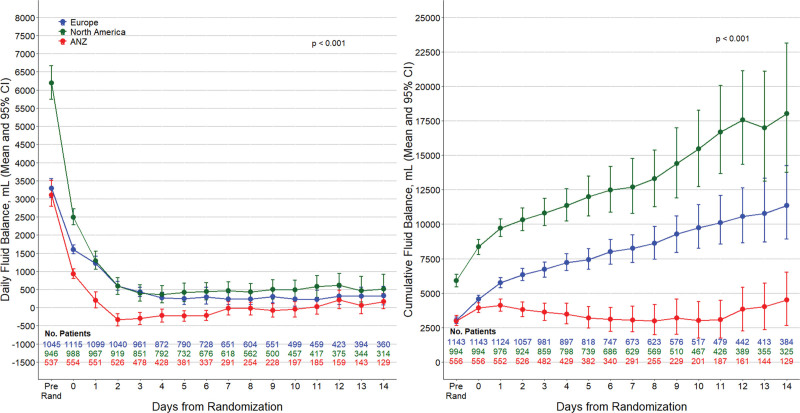
Daily and cumulative fluid balance according to geographical regions. ANZ = Australia and New Zealand.

Before randomization, the cumulative FB was highest among North American patients, with almost twice the value of European and ANZ patients, respectively (Table 1). Additionally, the cumulative FB from randomization or ICU admission was significantly more positive in North American and European patients than in ANZ patients (**Table 2** and **Fig. 2**). When medical and surgical patients were analyzed separately, the result remained the same (**eTable 3**, http://links.lww.com/CCX/B310). **eFigure 1** (http://links.lww.com/CCX/B310) presents the FB according to quintiles of SAPS II score in different regions and shows that ANZ patients had less positive FB than patients in other regions regardless of the severity of illness.

**TABLE 2.** Fluid Balance to a Maximum of 14 ICU Days From Randomization and From ICU Admissions Across Geographic Regions

Variable	North America (*n* = 994)	Europe (*n* = 1,143)	Australia and New Zealand (*n* = 556)	*p*
Fluid balance, mL				
Mean daily	283.9 (–342.1 to 1,158.5)	436.7 (–224.3 to 1,302.8)	–56.8 (–537.0 to 548.8)	< 0.001
Median daily	263.0 (–365.0 to 1,036.0)	432.5 (–235.0 to 1,299.0)	61.8 (–444.9 to 556.4)	< 0.001
Total	2,018.0 (–2,731.5 to 9,007.5)	2,900.0 (–1,932.5 to 9,179.0)	–493.0 (–4,342.2 to 3,458.0)	< 0.001
Fluid balance^a^, mL				
Mean daily	788.5 (94.9 to 1,790.6)	668.8 (28.9 to 1,597.4)	266.2 (–181.1 to 795.5)	< 0.001
Median daily	400.0 (–204.6 to 1,236.5)	548.8 (–80.0 to 1,365.4)	138.2 (–259.2 to 656.1)	< 0.001
Total	7,199.0 (700.5 to 16,129.5)	5,641.0 (157.0 to 13,713.5)	2,211.5 (–1,857.8 to 6,638.2)	< 0.001

a Including pre-randomization fluid balance.

Data are median (quartile 25th–quartile 75th) or *n* (%).

### Timing and Choice of Initial RRT Modality

In the standard strategy, time to RRT varied from a median of 30.7 hours in North America to 27.5 hours in ANZ and 48.0 hours in Europe (*p* < 0.001) (Table 1). In the accelerated strategy, the timing of initiation of RRT was clinically similar across regions.

Intermittent hemodialysis (IHD) was the initial RRT modality in 41.7% of European and 30.1% of North American patients, compared with only 0.7% of ANZ patients (Table 1). The initial use of continuous RRT (CRRT) was essentially the inverse. Use of sustained low-efficiency daily dialysis was infrequent and primarily confined to North America.

### Unadjusted Patient Outcomes According to Geographic Regions

There were significant differences in unadjusted 90-day mortality among regions (**Table 3**). In ANZ centers, ICU and hospital mortality were lower, and ICU, hospital, RRT, and ventilator-free days were greater compared with North American and European sites, respectively. Additionally, ANZ patients had the lowest proportion of dependence on RRT at 90 days and fewer patients with the combined outcome of death or RRT dependence at day 90 (Table 3). These observations were consistent when the cohort was stratified by allocation to accelerated or standard RRT initiation (**eFigs. 2** and **3**, http://links.lww.com/CCX/B310).

**TABLE 3.** Unadjusted Clinical Outcomes Stratified by Geographic Region

Outcome	North America (*n* = 994)	Europe (*n* = 1143)	Australia and New Zealand (*n* = 556)	*p*
90-d mortality	456/994 (45.9)	514/1143 (45.0)	177/556 (31.8)	< 0.001
Hospital outcomes				
ICU mortality	364/994 (36.6)	389/1143 (34.0)	125/556 (22.5)	< 0.001
Hospital mortality	421/994 (42.4)	461/1142 (40.4)	154/549 (28.1)	< 0.001
ICU-free days at day 28	3.5 (0.0–19.0)	4.0 (0.0–20.0)	16.0 (0.0–22.0)	< 0.001
Hospital-free days at day 90	0.0 (0.0–60.8)	4.0 (0.0–62.0)	50.0 (0.0–71.0)	< 0.001
Ventilator-free days at day 28	9.0 (0.0–22.0)	10.0 (0.0–24.0)	20.0 (0.0–25.0)	< 0.001
Renal outcomes				
RRT dependence at day 90	55/529 (10.4)	49/622 (7.9)	12/379 (3.2)	< 0.001
Death or RRT dependence at day 90	511/985 (51.9)	563/1136 (49.6)	189/556 (34.0)	
RRT-free days at day 90	8.5 (0.0–85.0)	59.0 (0.0–89.0)	82.0 (0.0–88.0)	< 0.001
Death category				0.011
Cardiovascular	288/455 (63.3)	282/510 (55.3)	116/172 (67.4)	
Metabolic	60/455 (13.2)	65/510 (12.7)	18/172 (10.5)	
Neurologic	18/455 (4.0)	35/510 (6.9)	12/172 (7.0)	
Respiratory	89/455 (19.6)	128/510 (25.1)	26/172 (15.1)	

RRT = renal replacement therapy.

Data are median (quartile 25th–quartile 75th) or *n* (%).

### Adjusted Patient Outcomes According to Geographic Regions

On univariable analysis, the differences between North America and Europe were confined to RRT-free days at 90 days, which were significantly higher in European patients. Compared with North America and Europe, ANZ patients experienced more favorable outcomes across all measures (**eTable 4**, http://links.lww.com/CCX/B310).

In the multivariable model, adjusted for age, sex, SAPS II score, admission type, and sepsis, there were no differences in outcomes between North America and Europe (**Table 4**). In contrast, there remained significant differences between patients in ANZ, who had better outcomes, compared with North America. The same pattern was observed when ANZ patients were compared with European patients, except there was no difference in RRT dependence at day 90. Additional analyses after adjustment for multiple statistically imbalanced baseline variables, including cumulative FB at randomization are reported in **eTable 5** (http://links.lww.com/CCX/B310).

**TABLE 4.** Multivariable Models for Key Outcomes

Outcome	North America vs. Europe		ANZ vs. Europe		ANZ vs. North America	
	Effect Estimate (95% CI)	*p*	Effect Estimate (95% CI)	*p*	Effect Estimate (95% CI)	*p*
90-d mortality (RD)	–0.37 (–6.32 to 5.60)	0.904	–11.31 (–17.74 to –4.85)	0.001	–10.27 (–17.47 to –3.07)	0.007
ICU mortality (RD)	–0.18 (–5.61 to 5.26)	0.949	–11.63 (–17.69 to –5.57)	< 0.001	–11.66 (–18.02 to –5.29)	0.001
Hospital mortality (RD)	0.39 (–5.25 to 6.05)	0.892	–10.96 (–16.95 to –4.97)	0.001	–10.98 (–17.92 to –4.06)	0.003
RRT dependence at day 90 (RD)	2.81 (–1.76 to 7.39)	0.234	–4.61 (–9.72 to 0.52)	0.083	–7.85 (–11.38 to –4.31)	< 0.001
Death or RRT dependence at day 90 (RD)	–0.34 (–6.36 to 5.69)	0.911	–11.56 (–18.10 to –4.99)	0.001	–10.50 (–17.74 to –3.24)	0.006
ICU-free days at day 28 (MD)	0.51 (–1.40 to 2.43)	0.599	5.86 (3.40–8.33)	< 0.001	4.72 (1.82–7.63)	0.001
Hospital-free days at day 90 (MD)	–0.59 (–6.54 to 5.36)	0.846	18.38 (9.74–27.03)	< 0.001	17.27 (7.12–27.43)	0.001
Ventilator-free days at day 28 (MD)	0.31 (–2.28 to 2.91)	0.812	6.27 (3.41–9.14)	< 0.001	5.72 (2.53–8.91)	< 0.001
RRT-free days at day 90 (MD)	–5.03 (–15.41 to 5.35)	0.342	18.74 (5.94–31.53)	0.004	25.67 (8.94–42.41)	0.003

ANZ = Australia and New Zealand, MD = median difference, RD = risk difference, RRT = renal replacement therapy.

RD calculated from a multivariable generalized linear model with binomial distribution and identity link.

MD calculated from a multivariable median regression using an interior point algorithm.

All models adjusted for age, sex, Simplified Acute Physiology Score II, type of admission (surgical vs. medical), and presence of sepsis. Sites were entered as random effect.

### Sensitivity Analyses

In sensitivity analysis among medical patients only, the key outcomes were aligned with those of the main analysis (**eTables 6** and **7**, http://links.lww.com/CCX/B310). Furthermore, findings were similar in analyses excluding the cardiac surgical patients (**eTables 8** and **9**, http://links.lww.com/CCX/B310) and after excluding all elective surgical patients (**eTables 10** and **11**, http://links.lww.com/CCX/B310).

Additional analyses according to allocation to the accelerated and standard strategy arms replicated the patterns seen in the entire cohorts (**eTables 12** and **13**, http://links.lww.com/CCX/B310).

Finally, we found French patients commenced IHD more frequently than other European patients (**eTable 14**, http://links.lww.com/CCX/B310) and had a significantly more positive FB than non-French European patients (**eTable 15**, http://links.lww.com/CCX/B310). Additionally, we found no significant outcome differences for French vs. non-French European patients when a clinical model was applied (**eTable 16**, http://links.lww.com/CCX/B310). In an alternative statistically derived model, however, differences in mortality in ICU, hospital, and 90 days, along with ventilator-free, ICU-free, and hospital-free days were evident (**eTable 17**, http://links.lww.com/CCX/B310).

## DISCUSSION

### Key Findings

In a post hoc secondary analysis of the STARRT-AKI trial, we found significant differences in patient characteristics, management, and RRT provision across three major geographic regions. Patients treated in North American and European centers had a more positive FB and were more likely to receive IHD as the initial modality of RRT, compared with those treated in ANZ centers. After adjusting for differences in predefined baseline characteristics, ANZ patients had better outcomes than those in North America and Europe, including a significantly lower 90-day mortality. The results of sensitivity analyses of the subgroup of medical patients, and subgroups excluding cardiac surgical and elective surgical patients aligned with those of the main analysis. However, there remained further variation within these geographic regions, likely due to aspects of both ICU and RRT organization (e.g., case-mix, ICU organization, staffing models, multidisciplinary teams) that differ between countries. For example, adverse patient outcomes have been associated with higher patient-to-nurse ratios, including adverse events, length of stay, and risk-adjusted mortality (24, 25). The availability of ICU beds and trained critical care personnel has also been associated with patient outcomes (26).

### Relationship to Previous Studies

Regional variations in the baseline characteristics of patients with AKI in general and those admitted to the ICU and either treated with RRT or characterized by stage 2 or 3 AKI have been reported for over 2 decades (10–12). These differences likely reflect regional differences in population health and disease characteristics, regional variation in ICU admission criteria, utilization and resources, and differences in the access to and organization of ICU services. Our findings expand such previous observations. Furthermore, regional differences have been reported for other aspects of critical care, including ventilation mode (27, 28) and the use of vasopressors and fluid resuscitation in septic patients (29).

The potential adverse consequences of fluid accumulation in critically ill patients in general, in patients receiving RRT, or those with severe AKI have been described (30–33). In aggregate, these studies have shown a less positive FB among patients treated with CRRT and those treated in ANZ compared with North America (13, 14). We reported consistently lower daily cumulative FB among both medical and surgical patients treated in ANZ compared with European or North American patients, extending previous observations about regional fluid management practices. Of note, another secondary analysis of the STARRT-AKI trial found that accelerated RRT initiation did not confer benefit in 90-day mortality among those with marked fluid accumulation compared with standard strategy, emphasizing that regional practices for fluid administration may be a more important determinant of fluid accumulation (34).

Regional variation in the timing and choice of initial RRT modality and the duration of its application have been characterized (35–38) and recently highlighted by a comparison of data from the two key randomized trials of RRT intensity (13, 14). The findings of our study confirm and extend the observation that there is a preferential use of CRRT as the modality of first choice in ANZ compared with an approach that combines IHD and CRRT in North America and Europe. Recently, another post hoc secondary analysis of the STARRT-AKI trial confirmed that initial use of CRRT, compared with IHD, was associated with a reduction in the composite outcome of death or dialysis dependence at 90 days (39).

A unique feature of this secondary analysis compared with previous comparative studies is that all patients were recruited and randomized within the same randomized trial using the same eligibility criteria. Thus, their baseline characteristics before randomization were documented in detail, and evaluation of the association between world regions and outcomes could be adjusted for baseline characteristics. Significant regional differences in practice and outcomes remained, after adjustment for baseline features, which is an important finding in the setting of a large international RCT where the standard-strategy arm was not strictly protocolized.

### Implications of Study Findings

Our study found that within the context of the STARRT-AKI trial, there were major regional variations in the characteristics of critically ill patients considered for RRT. Furthermore, such differences were associated with differences in achieved FB during the first 2 weeks of management in the ICU and the timing and choice of the initial modality of RRT. Finally, after adjustment and within the limitations of the available data, our analysis suggests that FB and RRT practice styles may be associated with outcomes. These findings should be considered when designing future trials investigating fluid management and RRT related aspects.

### Study Strengths and Limitations

This study has several strengths. First, the data were obtained from the largest randomized study of RRT in ICU conducted to date. Such broad representation from centers worldwide bolsters the generalizability of our findings. Second, data were rigorously collected and reviewed using explicit criteria. Baseline characteristics were collected in detail, thus minimizing ascertainment bias. Third, randomization was concealed, minimizing selection bias. Fourth, all patients’ inclusion criteria were standardized, minimizing indication bias. Finally, follow-up was rigorous and independent of treatment allocation or choice of RRT modality or FB achieved, thus minimizing performance bias.

We acknowledge several limitations. This post hoc observational analysis of data from a randomized trial is susceptible to the known limitations of such studies, including the inability to draw inferences about causation. First, the geographic regions selected were arbitrary and based on simple geographic proximity. We recognize there is likely significant variation between specific countries (3, 8, 13, 14), between centers in countries and even within individual centers; however, our objective with this secondary analysis was to provide a high-level description of practice variation within the context of a large international randomized trial. Furthermore, we recognize that enrollment contributions between countries (and centers) were from a broad diversity of hospitals and were also variable. This may limit inferences in circumstances where countries were represented by few centers enrolling many patients or vice versa. Second, we only captured data on FB in the trial through the first 14 days (34). Further, while we recorded data on FB at trial randomization and follow-up, we did not collect information on additional factors that may have influenced FB, notably the nature of fluid intake and output and the use of diuretics. Furthermore, FB does not reflect intravascular volume status and may not accurately reflect organ edema. In an observational study like ours, the association between a particular regional style of practice (less positive FB and greater use of CRRT) with better outcomes is only hypothesis-generating. Furthermore, the study centers from a given region may not necessarily reflect similar patient case-mix (i.e., sepsis, post-surgical) or management styles in all or even most ICUs in that region and selected regions are represented by variable sample sizes in our analysis. Yet, the clinical importance of the differences observed by regions both support the concept that regional practice variation is real and associates with patient outcomes. In our study, adjustments were made for key predefined baseline characteristics; however, many residual unmeasured confounders likely remain, which may impact our findings. The outcomes and FB may be driven by differences in patient characteristics and variables not recorded at baseline in the study. Such factors could plausibly include differences in how critical care is organized and provided across regions (e.g., variation in nurse-to-patient ratios). Furthermore, we only assessed the choice of initial RRT modality and could not describe the complexity of subsequent management. In this regard, shifts in FB and transitions in RRT modality reflect changes in patients’ conditions and medical responses thereto, such that FB and RRT modality may be markers, rather than drivers, of patient outcomes.

## CONCLUSIONS

In a post hoc secondary analysis of a large international randomized trial comparing accelerated vs. standard RRT initiation in critically ill patients with AKI, we observed differences in the crude and severity of illness adjusted outcomes and management styles across geographical regions regarding FB, initial RRT modality, and in the standard-strategy arm of the trial, the time to starting RRT. Considering these findings, this hypothesis-generating study provides the rationale and justification for randomized controlled trials that assess the impact of a less positive FB using a CRRT-predominant approach.

## Supplementary Material


