# Supplementary material for: Regional Practice Variation and Outcomes in the Standard Versus Accelerated Initiation of Renal Replacement Therapy in Acute Kidney Injury (STARRT-AKI) Trial: A Post Hoc Secondary Analysis
Source: Crit Care Explor. 2024 Feb 19;6(2):e1053. doi: 10.1097/CCE.0000000000001053 (PMC10878545; doi:10.1097/CCE.0000000000001053)
Supplement: Supplementary file 1 [file cc9-6-e1053-s001.pdf]

Regional Practice Variation and Outcomes in the STARRT-AKI trial: A Post-hoc Secondary Analysis

ONLINE SUPPLEMENT

Index

Additional Methods 2
eTable1 - Study sites and ethics approvals. 3
eTable 2 - Additional Baseline Characteristics 7
eTable 3 - Fluid Balance in the First 14 Days Across Geographic Regions in the Medical and Surgical Patient Subgroups. 9
eTable 4 - Univariable Models for Key Outcomes 10
eTable 5 - Full Multivariable Models for Key Outcomes 11
eTable 6 - Clinical Outcomes of Medical Patient Subgroup 12
eTable 7 - Multivariable Models for Key Outcomes of Medical Patient Subgroup 13
eTable 8- Clinical Outcomes with Cardiac Surgery Patients Excluded 14
eTable 9 - Multivariable Models for Key Outcomes with Cardiac Surgery Patients Excluded 15
eTable 10 - Clinical Outcomes with Elective Surgical Patients Excluded 16
eTable 11 - Multivariable Models for Key Outcomes with Elective Surgical Patients Excluded 17
eTable 12 - Full Multivariable Models for Key Outcomes (Accelerated-Strategy Arm) 18
eTable 13 - Full Multivariable Models for Key Outcomes (Standard-Strategy Arm) 19
eTable 14 - Summary of baseline characteristics between France and the rest of Europe 20
eTable 15 - Fluid Balance in the First 14 Days (France vs. Rest of Europe) 22
eTable 16 - Multivariable Models for Key Outcomes (France vs. rest of Europe) 23
eTable 17 - Full Multivariable Models for Key Outcomes (France vs. rest of Europe) 24
eFigure1 - Fluid balance accross regions according to severity of illness 25
eFigure 2 - Kaplan-Meier Curves of 90-Day Survival According to Geographic Region and Randomization Arm 26
eFigure 3 - Renal Replacement Therapy-Free Days According to Geographic Region 27
STARRT-AKI Investigators 28

Additional Methods

Normality of the data

Based on the central limit theorem, with a sample size of >2500 patients, we can assume that the distribution of sample means approximates a normal distribution regardless of the population's distribution. In addition, for the analyses of continuous variables we used median regression that is less impacted of non-normality of the data.

Selection of variables for sensitivity analyses

As a sensitivity analysis, an additional multivariable model included all variables with a P<0.05 at baseline and study site as a random effect. However, premorbid urine analysis and pre-randomization positive end-expiratory pressure (PEEP) were not included due to a high degree of missing data. Additionally, the initial RRT modality was not included because this was an exposure of interest that could explain the practice differences. Also, pre-randomization mechanical ventilation was not included because it was systematically missing in one specific country (missing not at random).

eTable1 - Study sites and ethics approvals.

CANADA		
**Site Name**	**Site Location**	**Ethics Approval**
University of Alberta Hospital	Alberta	Approved
Grey Nuns Community Hospital	Alberta	Covered under UofA
Mazankowski Alberta Heart Institute	Alberta	Covered under UofA
Misericordia Community Hospital	Alberta	Covered under UofA
Red Deer Regional Hospital	Alberta	Covered under UofA
Sturgeon Community Hospital	Alberta	Covered under UofA
Foothills Hospital	Alberta	Approved
Peter Lougheed Centre	Alberta	Approved
Royal Alexandra Hospital	Alberta	Approved
Fraser Health - Surrey Memorial Hospital	British Columbia	Approved
St. Paul's Hospital	British Columbia	Approved
Victoria General Hospital	British Columbia	Approved
Royal Jubilee Hospital	British Columbia	Approved
Health Sciences Centre	Manitoba	Approved
Lakeridge Health	Ontario	Approved
Hamilton General	Ontario	Approved
Health Sciences North	Ontario	Approved
Juravinski Hospital	Ontario	Approved
Kingston General Hospital	Ontario	Approved
London Health Sciences Centre – University Hospital	Ontario	Approved
London Health Sciences Centre – Victoria Hospital	Ontario	Approved
Mount Sinai Hospital	Ontario	Approved
St. Joseph's Healthcare	Ontario	Approved
St. Michael's Hospital	Ontario	Approved
Sunnybrook Health Sciences Centre	Ontario	Approved
The Ottawa Hospital - Civic Campus	Ontario	Approved
The Ottawa Hospital - General Campus	Ontario	
Toronto General Hospital - UHN	Ontario	Approved
Toronto Western Hospital - UHN	Ontario	Approved
Trillium Health Partners - Credit Valley Hospital	Ontario	Approved
Trillium Health Partners - Mississauga Hospital	Ontario	Approved
St. Joseph's Healthcare Toronto	Ontario	Approved
Centre Hospitalier Universitaire de Sherbrooke (CHUS)	Quebec	Approved
CHU de Québec (CHUQ) - Université Laval	Quebec	Approved
CHUM	Quebec	Approved
CHUM - Hotel-Dieu de Montreal	Quebec	Approved
CHUM - Notre Dame Hospital	Quebec	Approved
CIUSSS MCQ	Quebec	Approved
Hopital Maisonneuve-Rosemont	Quebec	Approved
Institut Universitaire de cardiologie et de pneumologie de Quebec (IUCPQ)	Quebec	Approved
McGill University Health Centre	Quebec	Approved
Regina Qu'Appelle Health Authority	Sasketchewan	Approved
Memorial University of Newfoundland	Newfoundland	Approved
**UNITED STATES**		
**Site Name**	**Site Location**	**Ethics Approval**
Mayo Clinic	Rochester	Approved
University of Alabama at Birmingham	Birmingham	Approved

University of Florida	Florida	Approved
University of Kentucky	Kentucky	Approved
Rhode Island Hospital	Rhode Island	Approved
The Miriam Hospital	Rhode Island	
University of California in Los Angeles	Los Angeles	Approved
**AUSTRALIA/NEW ZEALAND**		
**Site Name**	**Site Location**	**Ethics Approval**
Austin Hospital	Australia	Approved
Princess Alexandra Hospital	Australia	Approved
Western Health (Footscray Hospital)	Australia	Approved
The Alfred Hospital	Australia	Approved
Royal Prince Alfred Hospital	Australia	Approved
Nepean Hospital	Australia	
Sunshine Coast University Hospital (formerly Nambour)	Australia	Approved
Geelong Hospital	Australia	Approved
Bendigo Hospital	Australia	Approved
Ballarat Hospital	Australia	Approved
Eastern Hospital (Box Hill and Maroondah Hospital)	Australia	Approved
The Northern Hospital	Australia	Approved
Flinder Medical Centre	Australia	Approved
Concord Hospital	Australia	Approved
Royal North Shore Hospital	Australia	Approved
St. Vincent's Hospital	Australia	Approved
Wellington Hospital	New Zealand	Approved
Auckland City Hospital	New Zealand	Approved
Christchurch Hospital	New Zealand	Approved
Auckland Hospital DCCM	New Zealand	Approved
Hawke's Bay Hospital	New Zealand	Approved
Rotorua Hospital	New Zealand	Approved
Taranaki Hospital	New Zealand	Approved
Whangarei Hospital	New Zealand	Approved
Tauranga Hospital	New Zealand	Approved
**UNITED KINGDOM**		
**Site Name**	**Location**	**Ethics (Local) Approval**
Guy's and St. Thomas NHS Foundation	England	Approved
Queen's Medical Centre, Nottingham University Hospital	England	Approved
Buckinghamshire Healthcare: Stoke Mandeville Hospital	England	Approved
Buckinghamshire Healthcare: Wycombe Hospital	England	Approved
Milton Keynes Hospital	England	Approved
Leeds Teaching Hospital	England	Approved
East Kent University Hospitals NHS Trust	England	Approved
Lewisham and Greenwich NHS Trust - University Hospital Lewisham	England	Approved
Liverpool University Hospital	England	Approved
King's College Hospital	England	Approved
Warwick Hospital - South Warwickshire	England	Approved
Western Sussex Hospitals NHS Foundation Trust: St Richards Hospital	England	Approved
Western Sussex Hospitals NHS Foundation Trust: Worthing Hospital	England	Approved
St. Helens & Knowsley Teaching Hospital	England	Approved

York Teaching Hospital NHS Trust	England	Approved
Royal Bornemouth & Christchurch Hospitals NHS Trust	England	Approved
Lewisham and Greenwich NHS Trust - Queen Elizabeth Hospital	England	Approved
St. George's University Hospital	England	Approved
University Hospital of North Tees	England	Approved
Queen Elizabeth University Hospital	Scotland	Approved
Aberdeen Royal Infirmary (NHS Grampian)	Scotland	Approved
Golden Jubilee National Hospital (NHS Golden Jubilee)	Scotland	Approved
University Hospitals Coventry & Warwickshire	England	Approved
University Hospital Ayr (NHS Ayrshire and Arran)	Scotland	Approved
Hairmyres Hospital (NHS Lanarkshire)	Scotland	Approved
Royal Surrey County Hospital NHS Foundation Trust	England	Approved
Cardiff and Vale	England	Approved
Lincoln County Hospital	England	Approved
Sheffield	England	Approved
Monklands Hospital (NHS Lanarkshire)	Scotland	Approved
Royal Brompton and Harefield	England	Approved
Barnsley Hospital	England	Approved
Queen Elizabeth Hospital (Birmingham)	England	Approved
Barking, Havering and Redbridge University Hospital NHS	England	Approved
Chelsea and Westminster NHS Foundation Trust	England	Approved
**EUROPE**		
**Site Name**	**Site Location**	**Ethics Approval**
Medical University Innsbruck	Austria	Approved
Medical University Innsbruck, General and Surgical ICU	Austria	Approved
Medical University Graz	Austria	Approved
Medical University of Vienna	Austria	Approved
Ghent University Hospital	Belgium	Approved
CHU Brugmann University Hospital	Belgium	Approved
ZNA Hospital Antwerp	Belgium	Approved
Helsinki University Hospital	Finland	Approved
Tampere University Hospital	Finland	Under Helsinki
Turku University Hospital	Finland	Under Helsinki
University Hospital Munster	Germany	Approved
Klinikum Coburg	Germany	Approved
St. Vincent's University Hospital	Ireland	Approved
Centre Hospitalier Universitaire Vaudois (CHUV)	Switzerland	Approved
Geneva University Hospital	Switzerland	Approved
San Raffaele Hospital	Italy	Approved
Ospedale San Carlo	Italy	Approved
**FRANCE**		
**Site Name**		**Ethics Approval**
Hôpital Louis Mourier	France	Approved
Centre Hospitalier Départemental La Roche-Sur-Yon	France	Approved
CHU D'Amiens	France	Approved
Hôpital Pitiè Salpêtrière	France	Approved
Hôpital Avicenne	France	Approved
Hôpital Edouard Herriot	France	Approved
CH De Bourg-en-Bresse – Fleyriat	France	Approved
CHRU de Nîmes - Service de Réanimation	France	Approved

CHU De Rouen	France	Approved
CH Sud Francilien	France	Approved
CHU Dijon Bourgogne	France	Approved
CH Le Mans	France	Approved
Hotel Dieu - Service d'Anesthesie	France	Approved
CH de Béthune Beuvry – Germont et Gauthier	France	Approved
Hopital G. Montpied	France	Approved
CH de Dieppe	France	Approved
Hôpital Henri Mondor	France	Approved
Hôpital Civil	France	Approved
CHU de Pointe à Pitre	France	Approved
André Mignot	France	Approved
Centre Hospitalier Dr. Schaffner (Lens)	France	Approved
Hôpital Nord Laennec	France	Approved
Groupe Hospitalier Carnelle-Portes de l'Oise	France	Approved
Hotel Dieu – Service de Médicale	France	Approved
Hôpital de la Source - CHR d'Orléans	France	Approved
CH René DUBOS – Pontoise	France	Approved
CH Lyon Sud – Pierre Benite	France	Approved
HEGP : Hôpital Européen Georges-Pompidou	France	Approved
Service de reanimation medicale – Hopital Pitie Salpetriere	France	Approved

eTable 2 - Additional Baseline Characteristics

	North America (*n* = 994)	Europe (*n* = 1,143)	ANZ (*n* = 556)	*p* value
Age, years	65.4 (56.7 - 73.5)	68.4 (58.9 - 76.0)	66.7 (56.6 - 75.0)	< 0.001
Male gender - no. (%)	672 (67.6)	795 (69.6)	367 (66.0)	0.293
Weight, kg	89.0 (73.6 - 108.0)	82.0 (70.5 - 95.0)	87.0 (73.6 - 102.4)	< 0.001
SAPS II	62.0 (50.0 - 74.0)	55.0 (44.0 - 69.0)	61.0 (47.0 - 74.2)	< 0.001
Admission diagnosis - no. (%)				< 0.001
Cardiovascular	226 (22.8)	235 (20.6)	189 (34.1)	
Gastrointestinal/hepatic	185 (18.6)	149 (13.0)	109 (19.7)	
Hematologic	18 (1.8)	21 (1.8)	15 (2.7)	
Metabolic	33 (3.3)	57 (5.0)	8 (1.4)	
Neurologic	31 (3.1)	53 (4.6)	14 (2.5)	
Respiratory	181 (18.2)	273 (23.9)	70 (12.6)	
Septic	254 (25.6)	293 (25.6)	113 (20.4)	
Trauma	33 (3.3)	23 (2.0)	26 (4.7)	
Other	32 (3.2)	39 (3.4)	10 (1.8)	
Co-existing disorders - no. (%)				
Hypertension	601 (60.5)	641 (56.1)	286 (51.4)	0.002
Diabetes	353 (35.5)	324 (28.3)	150 (27.0)	< 0.001
Heart failure	139 (14.0)	173 (15.1)	58 (10.4)	0.026
Coronary artery disease	229 (23.0)	241 (21.1)	126 (22.7)	0.532
Liver disease	142 (14.3)	115 (10.1)	59 (10.6)	0.008
Risk factors for AKI - no. (%)				
Cardiopulmonary bypass	65 (6.5)	60 (5.2)	100 (18.0)	< 0.001
Aortic surgery	53 (5.3)	49 (4.3)	40 (7.2)	0.045
IV contrast	224 (22.6)	334 (29.3)	175 (31.5)	< 0.001
Aminoglycoside	37 (3.7)	211 (18.5)	49 (8.8)	< 0.001
Amphotericin B	7 (0.7)	8 (0.7)	6 (1.1)	0.634
Obstetric complications	4 (0.4)	3 (0.3)	1 (0.2)	0.813
Sepsis	619 (62.3)	664 (58.1)	305 (54.9)	0.013
Premorbid creatinine, µmol/L	96.0 (71.0 - 134.0)	96.0 (71.5 - 135.5)	96.0 (75.0 - 127.0)	0.996
eGFR, mL/min/1.73 m^2	65.4 (43.0 - 90.4)	65.1 (43.0 - 90.0)	66.0 (45.7 - 86.0)	0.711
Pre-randomization SOFA				
Total	13.0 (10.0 - 15.0)	11.0 (8.0 - 14.0)	12.0 (10.0 - 15.0)	< 0.001
Respiratory	2.5 (2.0 - 3.0)	2.0 (2.0 - 3.0)	2.0 (2.0 - 3.0)	0.291
Coagulation	1.0 (0.0 - 2.0)	0.0 (0.0 - 2.0)	1.0 (0.0 - 2.0)	< 0.001
Liver	0.0 (0.0 - 2.0)	0.0 (0.0 - 1.0)	0.0 (0.0 - 1.0)	0.005

eTable 2 - Additional Baseline Characteristics

	North America (*n* = 994)	Europe (*n* = 1,143)	ANZ (*n* = 556)	*p* value
Cardiovascular	3.0 (1.0 - 4.0)	4.0 (1.0 - 4.0)	4.0 (3.0 - 4.0)	0.002
Neurological	3.0 (2.0 - 4.0)	1.0 (0.0 - 4.0)	3.0 (1.0 - 4.0)	< 0.001
Renal	3.0 (2.0 - 4.0)	3.0 (2.0 - 3.0)	2.0 (2.0 - 3.0)	< 0.001

Data are median (quartile 25th - quartile 75th) or N (%).

Abbreviation: SOFA is Sequential Organ Failure Assessment; SAPS II is Simplified Acute Physiology Score II; AKI is acute kidney injury; eGFR is estimated glomerular filtration fraction; SOFA is Sequential Organ Failure Assessment; CFS is clinical frailty score; PEEP is positive end-expiratory pressure; RRT is renal replacement therapy.

eTable 3 - Fluid Balance in the First 14 Days Across Geographic Regions in the Medical and Surgical Patient Subgroups.

	Medical Patients				Surgical Patients			
	North America (*n* = 686)	Europe (*n* = 854)	ANZ (*n* = 267)	*p* value	North America (*n* = 308)	Europe (*n* = 289)	ANZ (*n* = 289)	*p* value
Fluid balance, mL								
Mean daily	337.0 (-298.8 - 1205.1)	497.7 (-189.4 - 1468.2)	-89.5 (-558.3 - 527.4)	< 0.001	122.4 (-419.7 - 969.2)	298.3 (-374.2 - 927.8)	-34.9 (-520.2 - 569.9)	0.002
Median daily	333.0 (-315.8 - 1147.0)	510.0 (-179.0 - 1444.5)	52.0 (-438.5 - 525.5)	< 0.001	108.5 (-515.2 - 784.6)	217.0 (-381.8 - 862.0)	100.5 (-461.0 - 579.5)	0.129
Total	2354.5 (-2588.5 - 9582.2)	3281.0 (-1383.8 - 9942.5)	-676.0 (-4586.5 - 3342.5)	< 0.001	781.0 (-3783.8 - 7388.2)	1986.0 (-3109.0 - 6870.0)	-310.0 (-4162 - 3603)	0.002
Fluid balance*, mL								
Mean daily	820.1 (130.5 - 1842.1)	695.9 (32.4 - 1711.4)	203.2 (-254.5 - 785.7)	< 0.001	724.1 (36.8 - 1692.0)	612.6 (26.3 - 1223.2)	305.0 (-132.9 - 798.1)	< 0.001
Median daily	466.5 (-173.5 - 1307.2)	630.0 (-49.8 - 1513.2)	119.0 (-261.8 - 646.5)	< 0.001	291.5 (-247.1 - 1062.2)	348.0 (-196.2 - 1010.2)	163.5 (-241.0 - 661.0)	0.094
Total	7608.5 (1135.0 - 16509.0)	5879.5 (164.5 - 14630.8)	2138.0 (-2332.0 - 6179.0)	< 0.001	6603.0 (340.2 - 15724.8)	5222.0 (109.0 - 11566.0)	2274.0 (-1103 - 6934)	< 0.001

Data are median (quartile 25th - quartile 75th) or N (%).

* Including pre-randomization fluid balance

eTable 4 - Univariable Models for Key Outcomes

	North America vs. Europe		ANZ vs. Europe		ANZ vs. North America	
	Effect Estimate (95% CI)	*p* value	Effect Estimate (95% CI)	*p* value	Effect Estimate (95% CI)	*p* value
90-day mortality (RD)	0.91 (-3.33 to 5.14)	0.675	-13.13 (-18.08 to -8.19)	< 0.001	-14.04 (-19.10 to -8.98)	< 0.001
ICU mortality (RD)	2.59 (-1.48 to 6.65)	0.212	-11.55 (-16.18 to -6.92)	< 0.001	-14.14 (-18.92 to -9.36)	< 0.001
Hospital mortality (RD)	1.99 (-2.20 to 6.18)	0.353	-12.32 (-17.18 to -7.45)	< 0.001	-14.30 (-19.30 to -9.31)	< 0.001
RRT dependence at day 90 (RD)	2.52 (-0.81 to 5.85)	0.138	-4.71 (-7.76 to -1.66)	0.002	-7.23 (-10.66 to -3.81)	< 0.001
Death or RRT dependence at day 90 (RD)	1.05 (-3.21 to 5.30)	0.629	-13.41 (-18.36 to -8.46)	< 0.001	-14.46 (-19.53 to -9.39)	< 0.001
ICU-free days at day 28 (MD)	-0.10 (-6.00 to 5.80)	0.973	12.00 (7.58 to 16.42)	< 0.001	12.14 (7.47 to 16.81)	< 0.001
Hospital-free days at day 90 (MD)	-4.00 (-16.05 to 8.05)	0.515	46.00 (32.61 to 59.39)	< 0.001	50.00 (41.83 to 58.17)	< 0.001
VFD at day 28 (MD)	-1.00 (-6.69 to 4.69)	0.730	10.00 (6.22 to 13.78)	< 0.001	11.00 (6.16 to 15.84)	< 0.001
RRT-free days at day 90 (MD)	-50.03 (-83.74 to -16.33)	0.004	23.00 (4.16 to 41.84)	0.017	73.07 (46.67 to 99.47)	< 0.001

Abbreviations: ICU is intensive care unit; RRT is renal replacement therapy; modified MAKE is major adverse kidney events inclusive of RRT at 90 days and mortality only; RD is risk difference; MD is median difference VFD is ventilator free days

Risk difference calculated from a univariable generalized linear model with binomial distribution and identity link.

Median difference calculated from a univariable median regression using an interior point algorithm.

eTable 5 - Full Multivariable Models for Key Outcomes

	North America vs. Europe		ANZ vs. Europe		ANZ vs. North America	
	**Effect Estimate (95% CI)**	***p* value**	**Effect Estimate (95% CI)**	***p* value**	**Effect Estimate (95% CI)**	***p* value**
90-day mortality (RD)	-5.77 (-12.11 to 0.58)	0.081	-11.16 (-17.80 to -4.53)	0.002	-6.63 (-14.33 to 1.08)	0.102
ICU mortality (RD)	-3.69 (-9.75 to 2.36)	0.242	-11.52 (-17.59 to -5.44)	0.001	-9.18 (-16.37 to -1.97)	0.017
Hospital mortality (RD)	-4.81 (-10.78 to 1.16)	0.122	-11.45 (-17.38 to -5.53)	0.001	-6.83 (-14.30 to 0.63)	0.083
RRT dependence at day 90 (RD)	3.16 (-2.15 to 8.47)	0.256	-4.33 (-9.62 to 1.02)	0.122	-7.79 (-12.03 to -3.59)	0.001
Death or RRT dependence at day 90 (RD)	-5.96 (-12.35 to 0.44)	0.074	-11.54 (-18.23 to -4.84)	0.002	-6.75 (-14.48 to 1.00)	0.098
ICU-free days at day 28 (MD)	1.35 (-0.70 to 3.41)	0.196	5.71 (3.94 to 7.48)	< 0.001	3.51 (0.88 to 6.14)	0.009
Hospital-free days at day 90 (MD)	3.05 (-3.03 to 9.12)	0.326	15.21 (8.56 to 21.87)	< 0.001	10.62 (1.23 to 20.01)	0.027
VFD days at day 28 (MD)	2.28 (-0.15 to 4.71)	0.066	5.20 (2.70 to 7.70)	< 0.001	3.74 (0.40 to 7.07)	0.028
RRT-free days at day 90 (MD)	-1.50 (-9.43 to 6.42)	0.710	16.15 (6.69 to 25.61)	0.001	21.35 (8.13 to 34.57)	0.002

Abbreviations: ICU is intensive care unit; RRT is renal replacement therapy; MAKE is major adverse kidney events; RD is risk difference; MD is median difference; VFD is ventilator free days

Risk difference calculated from a multivariable generalized linear model with binomial distribution and identity link.

Median difference calculated from a multivariable median regression using an interior point algorithm.

All models adjusted for age, weight, SAPS II, type of admission (surgical vs. medical), admission diagnosis, hypertension, diabetes, heart failure, liver disease, cardiopulmonary bypass, aortic surgery, IV contrast, aminoglycoside, presence of sepsis, premorbid eGFR, pre-randomization total SOFA, clinical frailty score, pre-randomization respiratory rate, pre-randomization cumulative fluid balance, pre-randomization pH, pre-randomization creatinine, pre-randomization hemoglobin, pre-randomization platelets, pre-randomization noradrenaline use, pre-randomization diuretic use. Sites were entered as random effect.

eTable 6 - Clinical Outcomes of Medical Patient Subgroup

	North America (n = 686)	Europe (n = 854)	ANZ (n = 267)	p value
90-day mortality - no. (%)	345 (50.3)	408 (47.8)	101 (37.8)	0.002
Hospital outcomes				
ICU mortality - no. (%)	272 (39.7)	314 (36.8)	68 (25.5)	< 0.001
Hospital mortality - no. (%)	320 (46.6)	368 / 853 (43.1)	87 / 261 (33.3)	0.001
ICU-free days at day 28	0.0 (0.0 - 18.0)	0.0 (0.0 - 20.0)	13.0 (0.0 - 21.5)	< 0.001
Hospital-free days at day 90	0.0 (0.0 - 57.0)	0.0 (0.0 - 62.0)	38.0 (0.0 - 68.0)	< 0.001
Ventilator-free days at day 28	5.0 (0.0 - 22.0)	7.0 (0.0 - 23.0)	18.0 (0.0 - 25.0)	< 0.001
Renal outcomes				
RRT dependence at day 90 - no. (%)	32 / 333 (9.6)	34 / 440 (7.7)	3 / 166 (1.8)	0.002
Death or RRT dependence at day 90 - no. (%)	345 / 678 (50.9)	408 / 848 (48.1)	101 / 267 (37.8)	0.001
RRT-free days at day 90	0.0 (0.0 - 82.0)	36.0 (0.0 - 89.0)	75.0 (0.0 - 88.0)	< 0.001
Death category - no. (%)				0.123
Cardiovascular	213 / 344 (61.9)	215 / 405 (53.1)	64 / 99 (64.6)	
Metabolic	46 / 344 (13.4)	56 / 405 (13.8)	10 / 99 (10.1)	
Neurological	14 / 344 (4.1)	29 / 405 (7.2)	5 / 99 (5.1)	
Respiratory	71 / 344 (20.6)	105 / 405 (25.9)	20 / 99 (20.2)	

Data are median (quartile 25th - quartile 75th) or N (%).

Abbreviation: ICU is intensive care unit; RRT is renal replacement therapy.

eTable 7 - Multivariable Models for Key Outcomes of Medical Patient Subgroup

	North America vs. Europe		ANZ vs. Europe		ANZ vs. North America	
	Effect Estimate (95% CI)	*p* value	Effect Estimate (95% CI)	*p* value	Effect Estimate (95% CI)	*p* value
90-day mortality (RD)	0.69 (-6.62 to 8.05)	0.854	-9.69 (-18.11 to -1.22)	0.030	-10.52 (-19.61 to -1.43)	0.027
ICU mortality (RD)	-0.76 (-7.35 to 5.85)	0.823	-12.25 (-20.15 to -4.33)	0.004	-12.35 (-20.61 to -4.05)	0.005
Hospital mortality (RD)	0.85 (-6.01 to 7.76)	0.809	-9.94 (-17.92 to -1.94)	0.019	-11.60 (-20.56 to -2.66)	0.014
RRT dependence at day 90 (RD)	2.43 (-2.73 to 7.58)	0.362	-5.79 (-11.26 to -0.31)	0.043	-8.07 (-13.16 to -2.98)	0.004
Death or RRT dependence at day 90 (RD)	0.81 (-6.63 to 8.31)	0.832	-10.08 (-18.70 to -1.40)	0.027	-10.99 (-20.15 to -1.83)	0.022
ICU-free days at day 28 (MD)	0.45 (-1.60 to 2.50)	0.667	4.46 (1.73 to 7.20)	0.001	3.60 (0.29 to 6.92)	0.033
Hospital-free days at day 90 (MD)	-1.22 (-8.79 to 6.35)	0.752	13.02 (2.86 to 23.18)	0.012	14.09 (-3.26 to 31.43)	0.112
Ventilator-free days at day 28 (MD)	1.49 (-1.75 to 4.74)	0.368	5.94 (3.05 to 8.84)	< 0.001	4.73 (0.95 to 8.50)	0.014
RRT-free days at day 90 (MD)	-3.95 (-15.97 to 8.07)	0.520	18.99 (5.66 to 32.33)	0.005	22.37 (-4.47 to 49.21)	0.103

Abbreviations: ICU is intensive care unit; RRT is renal replacement therapy; MAKE is major adverse kidney events; RD is risk difference; MD is median difference.

Risk difference calculated from a multivariable generalized linear model with binomial distribution and identity link.

Median difference calculated from a multivariable median regression using an interior point algorithm.

All models adjusted for age, sex, SAPS II, type of admission (surgical vs. medical), presence of sepsis and cumulative fluid balance pre-randomization. Sites were entered as random effect

eTable 8- Clinical Outcomes with Cardiac Surgery Patients Excluded

	**North America (*n* = 885)**	**Europe (*n* = 1050)**	**ANZ (*n* = 436)**	***p* value**
90-day mortality - no. (%)	416 (47.0)	481 (45.8)	142 (32.6)	< 0.001
Hospital outcomes				
ICU mortality - no. (%)	332 (37.5)	365 (34.8)	101 (23.2)	< 0.001
Hospital mortality - no. (%)	386 (43.6)	432 / 1049 (41.2)	124 / 429 (28.9)	< 0.001
ICU-free days at day 28	1.0 (0.0 - 19.0)	3.0 (0.0 - 20.0)	15.0 (0.0 - 22.0)	< 0.001
Hospital-free days at day 90	0.0 (0.0 - 59.0)	0.0 (0.0 - 62.0)	47.5 (0.0 - 70.0)	< 0.001
Ventilator-free days at day 28	8.0 (0.0 - 22.0)	9.0 (0.0 - 24.0)	19.0 (0.0 - 25.0)	< 0.001
Renal outcomes				
RRT dependence at day 90 - no. (%)	46 / 461 (10.0)	44 / 563 (7.8)	8 / 294 (2.7)	< 0.001
Death or RRT dependence at day 90 - no. (%)	416 / 877 (47.4)	481 / 1044 (46.1)	142 / 436 (32.6)	< 0.001
RRT-free days at day 90	0.0 (0.0 - 85.0)	54.0 (0.0 - 89.0)	81.0 (0.0 - 88.0)	< 0.001
Death category - no. (%)				0.071
Cardiovascular	259 / 415 (62.4)	262 / 478 (54.8)	93 / 140 (66.4)	
Metabolic	55 / 415 (13.3)	62 / 478 (13.0)	16 / 140 (11.4)	
Neurological	17 / 415 (4.1)	32 / 478 (6.7)	8 / 140 (5.7)	
Respiratory	84 / 415 (20.2)	122 / 478 (25.5)	23 / 140 (16.4)	

Data are median (quartile 25th - quartile 75th) or N (%).

Abbreviation: ICU is intensive care unit; RRT is renal replacement therapy.

eTable 9 - Multivariable Models for Key Outcomes with Cardiac Surgery Patients Excluded

	North America vs. Europe		ANZ vs. Europe		ANZ vs. North America	
	**Effect Estimate (95% CI)**	***p* value**	**Effect Estimate (95% CI)**	***p* value**	**Effect Estimate (95% CI)**	***p* value**
90-day mortality (RD)	-0.17 (-6.55 to 6.23)	0.958	-10.90 (-17.69 to -4.07)	0.003	-10.19 (-17.92 to -2.43)	0.012
ICU mortality (RD)	-0.25 (-6.24 to 5.75)	0.935	-10.84 (-17.34 to -4.29)	0.002	-11.24 (-18.26 to -4.19)	0.003
Hospital mortality (RD)	0.21 (-5.98 to 6.41)	0.948	-10.62 (-16.96 to -4.27)	0.002	-10.70 (-18.40 to -2.99)	0.008
RRT dependence at day 90 (RD)	3.08 (-1.81 to 7.99)	0.223	-4.73 (-9.74 to 0.32)	0.070	-8.23 (-12.39 to -4.06)	0.001
Death or RRT dependence at day 90 (RD)	-0.10 (-6.57 to 6.38)	0.975	-11.17 (-18.09 to -4.21)	0.003	-10.51 (-18.29 to -2.70)	0.010
ICU-free days at day 28 (MD)	0.75 (-1.41 to 2.90)	0.497	5.27 (3.04 to 7.49)	< 0.001	4.31 (1.47 to 7.15)	0.003
Hospital-free days at day 90 (MD)	-0.86 (-8.23 to 6.52)	0.820	16.49 (6.90 to 26.08)	0.001	17.19 (5.15 to 29.23)	0.005
Ventilator-free days at day 28 (MD)	1.37 (-1.78 to 4.51)	0.394	5.69 (3.00 to 8.37)	< 0.001	5.00 (1.48 to 8.53)	0.006
RRT-free days at day 90 (MD)	-4.01 (-15.48 to 7.45)	0.493	18.38 (7.04 to 29.72)	0.002	25.58 (6.44 to 44.71)	0.009

Abbreviations: ICU is intensive care unit; RRT is renal replacement therapy; MAKE is major adverse kidney events; RD is risk difference; MD is median difference.

Risk difference calculated from a multivariable generalized linear model with binomial distribution and identity link.

Median difference calculated from a multivariable median regression using an interior point algorithm.

All models adjusted for age, sex, SAPS II, type of admission (surgical vs. medical), presence of sepsis and cumulative fluid balance pre-randomization. Sites were entered as random effect.

eTable 10 - Clinical Outcomes with Elective Surgical Patients Excluded

	North America (n = 880)	Europe (n = 1035)	ANZ (n = 440)	p value
90-day mortality - no. (%)	420 (47.7)	477 (46.1)	149 (33.9)	< 0.001
Hospital outcomes				
ICU mortality - no. (%)	335 (38.1)	362 (35.0)	105 (23.9)	< 0.001
Hospital mortality - no. (%)	390 (44.3)	429 / 1034 (41.5)	130 / 433 (30.0)	< 0.001
ICU-free days at day 28	1.0 (0.0 - 19.0)	1.0 (0.0 - 20.0)	14.0 (0.0 - 22.0)	< 0.001
Hospital-free days at day 90	0.0 (0.0 - 59.0)	0.0 (0.0 - 62.0)	44.0 (0.0 - 71.0)	< 0.001
Ventilator-free days at day 28	8.0 (0.0 - 22.0)	8.0 (0.0 - 23.0)	19.0 (0.0 - 25.0)	< 0.001
Renal outcomes				
RRT dependence at day 90 - no. (%)	44 / 451 (9.8)	45 / 552 (8.2)	7 / 291 (2.4)	< 0.001
Death or RRT dependence at day 90 - no. (%)	420 / 871 (48.2)	477 / 1029 (46.4)	149 / 440 (33.9)	< 0.001
RRT-free days at day 90	0.0 (0.0 - 84.0)	51.0 (0.0 - 89.0)	80.0 (0.0 - 88.0)	< 0.001
Death category - no. (%)				0.011
Cardiovascular	265 / 419 (63.2)	257 / 473 (54.3)	97 / 146 (66.4)	
Metabolic	55 / 419 (13.1)	64 / 473 (13.5)	13 / 146 (8.9)	
Neurological	17 / 419 (4.1)	34 / 473 (7.2)	12 / 146 (8.2)	
Respiratory	82 / 419 (19.6)	118 / 473 (24.9)	24 / 146 (16.4)	

Data are median (quartile 25th - quartile 75th) or N (%).

Abbreviation: ICU is intensive care unit; RRT is renal replacement therapy.

eTable 11 - Multivariable Models for Key Outcomes with Elective Surgical Patients Excluded

	North America vs. Europe		ANZ vs. Europe		ANZ vs. North America	
	Effect Estimate (95% CI)	*p* value	Effect Estimate (95% CI)	*p* value	Effect Estimate (95% CI)	*p* value
90-day mortality (RD)	-0.31 (-6.85 to 6.24)	0.927	-9.17 (-16.50 to -1.83)	0.018	-8.72 (-16.75 to -0.65)	0.038
ICU mortality (RD)	-0.09 (-6.15 to 5.98)	0.978	-10.02 (-16.84 to -3.21)	0.006	-10.97 (-18.14 to -3.77)	0.004
Hospital mortality (RD)	0.38 (-5.88 to 6.64)	0.907	-9.10 (-15.85 to -2.36)	0.011	-9.90 (-17.80 to -1.98)	0.017
RRT dependence at day 90 (RD)	2.52 (-2.73 to 7.77)	0.353	-5.03 (-10.46 to 0.44)	0.075	-7.70 (-12.24 to -3.09)	0.003
Death or RRT dependence at day 90 (RD)	-0.25 (-6.89 to 6.39)	0.941	-9.46 (-16.91 to -1.99)	0.017	-9.05 (-17.13 to -0.92)	0.032
ICU-free days at day 28 (MD)	0.51 (-1.66 to 2.67)	0.647	4.73 (2.10 to 7.36)	< 0.001	4.13 (1.31 to 6.96)	0.004
Hospital-free days at day 90 (MD)	-1.05 (-7.83 to 5.73)	0.762	15.31 (5.13 to 25.48)	0.003	16.11 (2.83 to 29.39)	0.018
Ventilator-free days at day 28 (MD)	1.73 (-1.27 to 4.73)	0.259	5.55 (2.49 to 8.62)	< 0.001	4.73 (1.09 to 8.37)	0.011
RRT-free days at day 90 (MD)	-5.09 (-16.94 to 6.77)	0.400	17.34 (5.39 to 29.30)	0.005	22.17 (1.09 to 43.26)	0.039

Abbreviations: ICU is intensive care unit; RRT is renal replacement therapy; MAKE is major adverse kidney events; RD is risk difference; MD is median difference.

Risk difference calculated from a multivariable generalized linear model with binomial distribution and identity link.

Median difference calculated from a multivariable median regression using an interior point algorithm.

All models adjusted for age, sex, SAPS II, type of admission (surgical vs. medical), presence of sepsis and cumulative fluid balance pre-randomization. Sites were entered as random effect.

eTable 12 - Full Multivariable Models for Key Outcomes (Accelerated-Strategy Arm)

	North America vs. Europe		ANZ vs. Europe		ANZ vs. North America	
	Effect Estimate (95% CI)	*p* value	Effect Estimate (95% CI)	*p* value	Effect Estimate (95% CI)	*p* value
90-day mortality (RD)	-5.83 (-13.85 to 2.19)	0.166	-10.66 (-19.56 to -1.74)	0.027	-5.65 (-15.93 to 4.61)	0.299
ICU mortality (RD)	-4.02 (-11.38 to 3.33)	0.299	-10.01 (-17.61 to -2.43)	0.016	-7.18 (-15.72 to 1.36)	0.119
Hospital mortality (RD)	-3.99 (-11.63 to 3.61)	0.316	-8.80 (-16.96 to -0.65)	0.047	-4.66 (-14.44 to 5.07)	0.367
RRT dependence at day 90 (RD)	5.08 (-3.34 to 13.37)	0.249	-5.96 (-14.12 to 2.21)	0.168	-11.14 (-18.35 to -3.90)	0.006
Death or RRT dependence at day 90 (RD)	-5.86 (-13.94 to 2.22)	0.167	-10.75 (-19.66 to -1.83)	0.025	-5.79 (-16.11 to 4.51)	0.289
ICU-free days at day 28 (MD)	0.91 (-1.43 to 3.25)	0.444	4.96 (2.38 to 7.53)	< 0.001	4.45 (0.73 to 8.17)	0.019
Hospital-free days at day 90 (MD)	2.16 (-5.26 to 9.59)	0.568	13.02 (1.57 to 24.47)	0.026	12.32 (-0.46 to 25.10)	0.059
VFD days at day 28 (MD)	0.81 (-1.92 to 3.54)	0.562	4.97 (1.80 to 8.14)	0.002	5.15 (1.22 to 9.08)	0.010
RRT-free days at day 90 (MD)	-5.15 (-15.39 to 5.08)	0.324	18.60 (6.67 to 30.54)	0.002	24.74 (3.63 to 45.85)	0.022

Abbreviations: ICU is intensive care unit; RRT is renal replacement therapy; MAKE is major adverse kidney events; RD is risk difference; MD is median difference; VFD is ventilator free days

Risk difference calculated from a multivariable generalized linear model with binomial distribution and identity link.

Median difference calculated from a multivariable median regression using an interior point algorithm.

All models adjusted for age, weight, SAPS II, type of admission (surgical vs. medical), admission diagnosis, hypertension, diabetes, heart failure, liver disease, cardiopulmonary bypass, aortic surgery, IV contrast, aminoglycoside, presence of sepsis, premorbid eGFR, pre-randomization total SOFA, clinical frailty score, pre-randomization respiratory rate, pre-randomization cumulative fluid balance, pre-randomization pH, pre-randomization creatinine, pre-randomization hemoglobin, pre-randomization platelets, pre-randomization noradrenaline use, pre-randomization diuretic use. Sites were entered as random effect.

eTable 13 - Full Multivariable Models for Key Outcomes (Standard-Strategy Arm)

	North America vs. Europe		ANZ vs. Europe		ANZ vs. North America	
	Effect Estimate (95% CI)	*p* value	Effect Estimate (95% CI)	*p* value	Effect Estimate (95% CI)	*p* value
90-day mortality (RD)	-5.24 (-13.71 to 3.23)	0.239	-11.27 (-20.18 to -2.36)	0.020	-7.92 (-17.03 to 1.21)	0.105
ICU mortality (RD)	-2.88 (-10.85 to 5.08)	0.491	-12.62 (-20.59 to -4.64)	0.004	-11.75 (-20.84 to -2.62)	0.017
Hospital mortality (RD)	-4.89 (-12.92 to 3.13)	0.247	-13.62 (-21.88 to -5.34)	0.003	-9.04 (-17.72 to -0.36)	0.053
RRT dependence at day 90 (RD)	-0.30 (-5.52 to 4.93)	0.915	-4.12 (-8.86 to 0.61)	0.113	-4.28 (-9.40 to 0.84)	0.118
Death or RRT dependence at day 90 (RD)	-5.55 (-14.03 to 2.92)	0.212	-11.89 (-20.78 to -3.00)	0.014	-7.91 (-17.06 to 1.26)	0.107
ICU-free days at day 28 (MD)	2.32 (-0.28 to 4.92)	0.080	6.97 (4.21 to 9.72)	< 0.001	3.44 (0.50 to 6.38)	0.022
Hospital-free days at day 90 (MD)	7.53 (-1.10 to 16.16)	0.088	15.62 (8.04 to 23.20)	< 0.001	7.18 (-3.11 to 17.47)	0.172
VFD days at day 28 (MD)	2.77 (-0.49 to 6.04)	0.096	5.67 (1.96 to 9.38)	0.003	2.60 (-1.77 to 6.96)	0.244
RRT-free days at day 90 (MD)	3.19 (-10.12 to 16.49)	0.639	13.56 (-0.59 to 27.72)	0.061	15.51 (-2.06 to 33.09)	0.084

Abbreviations: ICU is intensive care unit; RRT is renal replacement therapy; MAKE is major adverse kidney events; RD is risk difference; MD is median difference; VFD is ventilator free days

Risk difference calculated from a multivariable generalized linear model with binomial distribution and identity link.

Median difference calculated from a multivariable median regression using an interior point algorithm.

All models adjusted for age, weight, SAPS II, type of admission (surgical vs. medical), admission diagnosis, hypertension, diabetes, heart failure, liver disease, cardiopulmonary bypass, aortic surgery, IV contrast, aminoglycoside, presence of sepsis, premorbid eGFR, pre-randomization total SOFA, clinical frailty score, pre-randomization respiratory rate, pre-randomization cumulative fluid balance, pre-randomization pH, pre-randomization creatinine, pre-randomization hemoglobin, pre-randomization platelets, pre-randomization noradrenaline use, pre-randomization diuretic use. Sites were entered as random effect.

eTable 14 - Summary of baseline characteristics between France and the rest of Europe

	France (*n* = 747)	Rest of Europe (*n* = 396)	*p* value
Age, years	68.6 (60.4 - 76.1)	67.8 (56.3 - 75.9)	0.111
Male gender - no. (%)	527 (70.6)	268 (67.7)	0.311
Weight, kg	81.8 (70.0 - 95.0)	82.4 (71.8 - 95.0)	0.224
SAPS II	54.0 (44.0 - 68.0)	58.0 (44.8 - 70.0)	0.233
Randomization group - no. (%)			0.756
Accelerated arm	371 (49.7)	201 (50.8)	
Standard arm	376 (50.3)	195 (49.2)	
Hours between eligibility and RRT	8.4 (4.0 - 32.2)	9.6 (5.4 - 27.1)	0.301
Accelerated arm	5.1 (3.1 - 8.3)	6.4 (3.9 - 8.6)	0.030
Standard arm	63.7 (27.5 - 87.9)	33.3 (21.4 - 62.0)	< 0.001
Initial modality - no. (%)			< 0.001
CRRT	222 (40.2)	270 (86.0)	
IHD	325 (58.9)	36 (11.5)	
SLED	5 (0.9)	8 (2.5)	
Type of admission - no. (%)			< 0.001
Medical	609 (81.5)	245 (61.9)	
Scheduled surgery	52 (7.0)	56 (14.1)	
Unscheduled surgery	86 (11.5)	95 (24.0)	
Admission diagnosis - no. (%)			< 0.001
Cardiovascular	119 (15.9)	116 (29.3)	
Gastrointestinal/hepatic	89 (11.9)	60 (15.2)	
Hematologic	17 (2.3)	4 (1.0)	
Metabolic	44 (5.9)	13 (3.3)	
Neurologic	45 (6.0)	8 (2.0)	
Respiratory	188 (25.2)	85 (21.5)	
Septic	217 (29.0)	76 (19.2)	
Trauma	13 (1.7)	10 (2.5)	
Other	15 (2.0)	24 (6.1)	
Co-existing disorders - no. (%)			
Hypertension	441 (59.1)	200 (50.5)	0.006
Diabetes	221 (29.6)	103 (26.0)	0.215
Heart failure	134 (18.0)	39 (9.8)	< 0.001
Coronary artery disease	145 (19.4)	96 (24.2)	0.067
Liver disease	73 (9.8)	42 (10.6)	0.680
Risk factors for AKI - no. (%)			
Cardiopulmonary bypass	22 (2.9)	38 (9.6)	< 0.001
Aortic surgery	12 (1.6)	37 (9.3)	< 0.001
IV contrast	189 (25.3)	145 (36.8)	< 0.001
Aminoglycoside	161 (21.6)	50 (12.7)	< 0.001
Amphotericin B	6 (0.8)	2 (0.5)	0.721
Obstetric complications	3 (0.4)	0 (0.0)	0.555

eTable 14 - Summary of baseline characteristics between France and the rest of Europe

	France (n = 747)	Rest of Europe (n = 396)	p value
Sepsis	459 (61.4)	205 (51.8)	0.002
Premorbid creatinine, μmol/L	99.0 (73.0 - 141.0)	90.0 (70.7 - 123.8)	0.004
eGFR, mL/min/1.73 m^2	62.4 (40.6 - 88.6)	70.2 (47.9 - 92.1)	0.001
eGFR < 60, mL/min/1.73 m^2	390 (52.2)	244 (61.6)	0.003
Pre-randomization SOFA			
Total	11.0 (8.0 - 13.0)	12.0 (9.0 - 14.0)	< 0.001
Respiratory	2.0 (1.0 - 3.0)	3.0 (2.0 - 3.0)	< 0.001
Coagulation	0.0 (0.0 - 2.0)	0.0 (0.0 - 2.0)	0.615
Liver	0.0 (0.0 - 1.0)	0.0 (0.0 - 1.0)	0.689
Cardiovascular	4.0 (1.0 - 4.0)	4.0 (3.0 - 4.0)	0.092
Neurological	1.0 (0.0 - 3.0)	2.0 (0.0 - 4.0)	< 0.001
Renal	3.0 (2.0 - 4.0)	2.0 (2.0 - 3.0)	< 0.001
Pre-randomization CFS	2.0 (0.0 - 4.0)	2.0 (0.0 - 4.0)	0.515
Pre-randomization signs			
Respiratory rate, breaths/min	25.0 (20.0 - 30.0)	20.0 (15.0 - 24.0)	< 0.001
PEEP, cmH_2O	8.0 (5.0 - 10.0)	8.0 (7.0 - 10.0)	< 0.001
Cumulative fluid balance, mL	2223 (699 - 4283)	2777 (887 - 5089)	0.049
Pre-randomization blood tests			
pH	7.33 (7.26 - 7.39)	7.34 (7.28 - 7.38)	0.707
Creatinine, μmol/L	275.0 (203.5 - 369.5)	252.5 (194.4 - 335.2)	0.009
Hemoglobin, g/L	105.0 (88.0 - 120.0)	96.0 (84.0 - 115.2)	< 0.001
Platelets, x10^9/L	168.0 (96.0 - 254.0)	162.0 (97.0 - 245.0)	0.554
Pre-randomization support			
Mechanical ventilation or CPAP - no. (%)	564 (75.5)	300 (75.8)	0.942
Noradrenaline use - no. (%)	442 (59.2)	284 (71.7)	< 0.001
Dose, μg/kg/min	0.46 (0.20 - 1.00)	0.17 (0.10 - 0.31)	< 0.001
Diuretic use - no. (%)	137 (18.3)	152 (38.4)	< 0.001

Data are median (quartile 25th - quartile 75th) or N (%).

Abbreviation: SOFA is Sequential Organ Failure Assessment; RRT is renal replacement therapy; SAPS II is Simplified Acute Physiology Score II; CRRT is continuous renal replacement therapy; IHD is intermittent hemodialysis; SLED is slow low efficiency daily dialysis; AKI is acute kidney injury; eGFR is estimated glomerular filtration fraction; SOFA is Sequential Organ Failure Assessment; CFS is clinical frailty score; PEEP is positive end-expiratory pressure; RRT is renal replacement therapy.

eTable 15 - Fluid Balance in the First 14 Days (France vs. Rest of Europe)

	France (*n* = 747)	Rest of Europe (*n* = 396)	*p* value
Fluid balance, mL			
Mean daily	645.0 (-64.2, 1601.4)	139.7 (-388.2, 743.5)	< 0.001
Median daily	680.0 (-75.0, 1570.5)	83.0 (-435.0, 634.1)	< 0.001
Total	4100.0 (-429.5, 12313.0)	1101.5 (-3306.2, 5666.8)	< 0.001
Fluid balance*, mL			
Mean daily	845.6 (138.9, 1861.3)	419.2 (-48.9, 1053.0)	< 0.001
Median daily	781.8 (58.6, 1699.6)	175.0 (-343.4, 746.9)	< 0.001
Total	6557.0 (809.0, 15945.5)	3613.5 (-523.5, 10040.0)	< 0.001

Data are median (quartile 25th - quartile 75th) or N (%).

* Including pre-randomization fluid balance

eTable 16 - Multivariable Models for Key Outcomes (France vs. rest of Europe)

	France (*n* = 747)	Rest of Europe (*n* = 396)	Effect Estimate (95% CI)	*p* value
90-day mortality (RD)	367 / 747 (49.1)	147 / 396 (37.1)	10.38 (2.78 to 18.07)	0.011
ICU mortality (RD)	282 / 747 (37.8)	107 / 396 (27.0)	9.55 (2.43 to 16.70)	0.012
Hospital mortality (RD)	330 / 746 (44.2)	131 / 396 (33.1)	9.64 (2.65 to 16.70)	0.011
RRT dependence at day 90 (RD)	30 / 376 (8.0)	19 / 246 (7.7)	1.08 (-5.60 to 7.81)	0.754
Death or RRT dependence at day 90 (RD)	397 / 743 (53.4)	166 / 393 (42.2)	10.30 (2.52 to 18.17)	0.013
ICU-free days at day 28 (MD)	0.0 (0.0 - 20.0)	8.0 (0.0 - 22.0)	-1.13 (-3.71 to 1.46)	0.393
Hospital-free days at day 90 (MD)	0.0 (0.0 - 60.0)	28.0 (0.0 - 65.2)	-5.43 (-14.50 to 3.64)	0.241
Ventilator-free days at day 28 (MD)	5.0 (0.0 - 23.8)	13.5 (0.0 - 24.0)	-1.08 (-4.15 to 2.00)	0.493
RRT-free days at day 90 (MD)	0.0 (0.0 - 89.0)	73.0 (0.0 - 88.0)	-12.43 (-26.79 to 1.93)	0.090

Abbreviations: ICU is intensive care unit; RRT is renal replacement therapy; MAKE is major adverse kidney events; RD is risk difference; MD is median difference.

Risk difference calculated from a multivariable generalized linear model with binomial distribution and identity link.

Median difference calculated from a multivariable median regression using an interior point algorithm.

All models adjusted for age, sex, SAPS II, type of admission (surgical vs. medical) and presence of sepsis. Sites were entered as random effect. Reference is Europe

eTable 17 - Full Multivariable Models for Key Outcomes (France vs. rest of Europe)

	France (*n* = 747)	Rest of Europe (*n* = 396)	Effect Estimate (95% CI)	*p* value
90-day mortality (RD)	367 / 747 (49.1)	147 / 396 (37.1)	12.22 (4.46 to 19.96)	0.004
ICU mortality (RD)	282 / 747 (37.8)	107 / 396 (27.0)	11.17 (3.82 to 18.51)	0.006
Hospital mortality (RD)	330 / 746 (44.2)	131 / 396 (33.1)	11.53 (4.69 to 18.31)	0.003
RRT dependence at day 90 (RD)	30 / 376 (8.0)	19 / 246 (7.7)	1.69 (-5.71 to 9.06)	0.665
Death or RRT dependence at day 90 (RD)	397 / 743 (53.4)	166 / 393 (42.2)	12.24 (4.38 to 20.09)	0.004
ICU-free days at day 28 (MD)	0.0 (0.0 - 20.0)	8.0 (0.0 - 22.0)	-3.49 (-5.77 to -1.21)	0.003
Hospital-free days at day 90 (MD)	0.0 (0.0 - 60.0)	28.0 (0.0 - 65.2)	-8.39 (-16.46 to -0.31)	0.042
Ventilator-free days at day 28 (MD)	5.0 (0.0 - 23.8)	13.5 (0.0 - 24.0)	-3.95 (-6.63 to -1.28)	0.004
RRT-free days at day 90 (MD)	0.0 (0.0 - 89.0)	73.0 (0.0 - 88.0)	-15.37 (-28.01 to -2.73)	0.017

Abbreviations: ICU is intensive care unit; RRT is renal replacement therapy; MAKE is major adverse kidney events; RD is risk difference; MD is median difference.

Risk difference calculated from a multivariable generalized linear model with binomial distribution and identity link.

Median difference calculated from a multivariable median regression using an interior point algorithm.

All models adjusted for age, weight, SAPS II, type of admission (surgical vs. medical), admission diagnosis, hypertension, diabetes, heart failure, liver disease, cardiopulmonary bypass, aortic surgery, IV contrast, aminoglycoside, presence of sepsis, premorbid eGFR, pre-randomization total SOFA, clinical frailty score, pre-randomization respiratory rate, pre-randomization cumulative fluid balance, pre-randomization pH, pre-randomization creatinine, pre-randomization hemoglobin, pre-randomization platelets, pre-randomization noradrenaline use, pre-randomization diuretic use. Sites were entered as random effect

eFigure1 - Fluid balance accross regions according to severity of illness

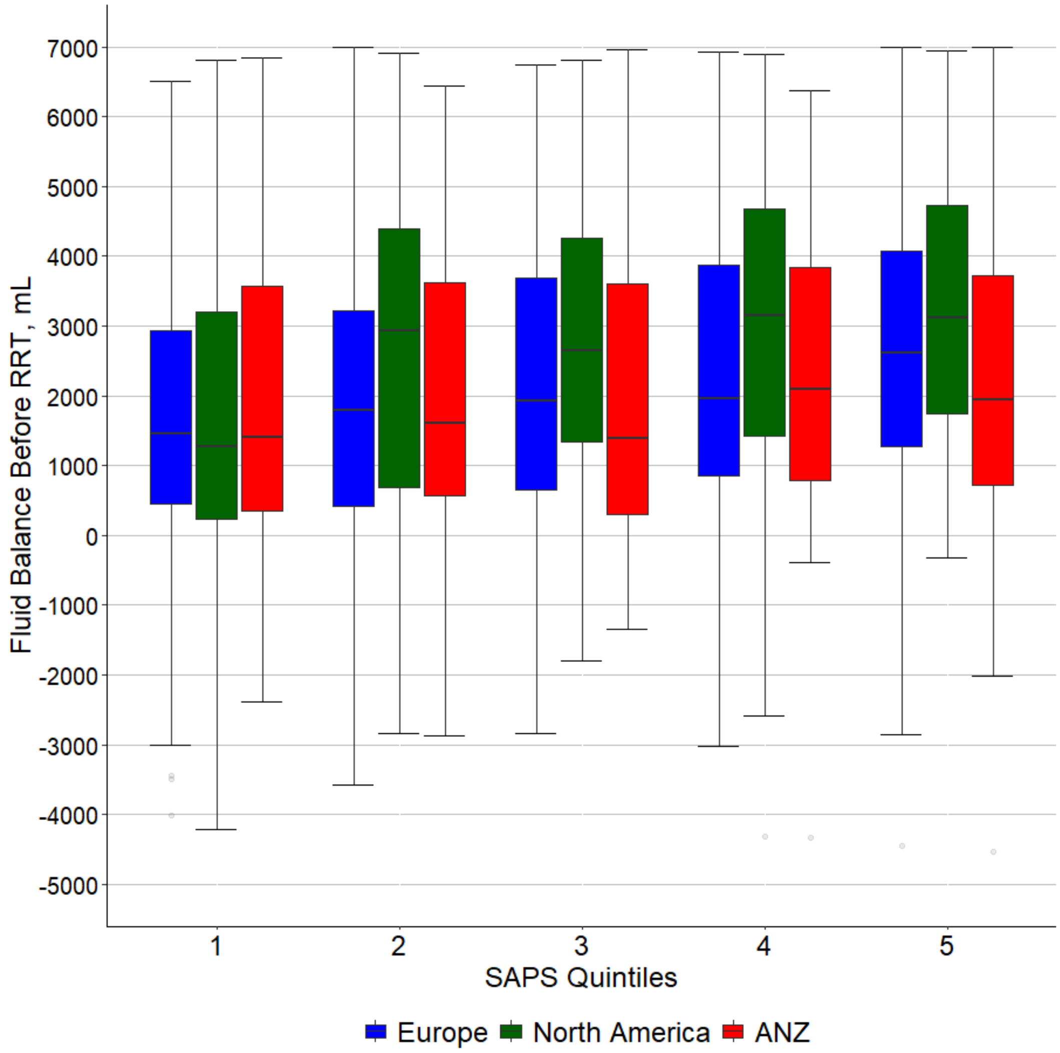

RRT: renal replacement therapy, SAPS: simplified acute physiology score

eFigure 2 - Kaplan-Meier Curves of 90-Day Survival According to Geographic Region and Randomization Arm

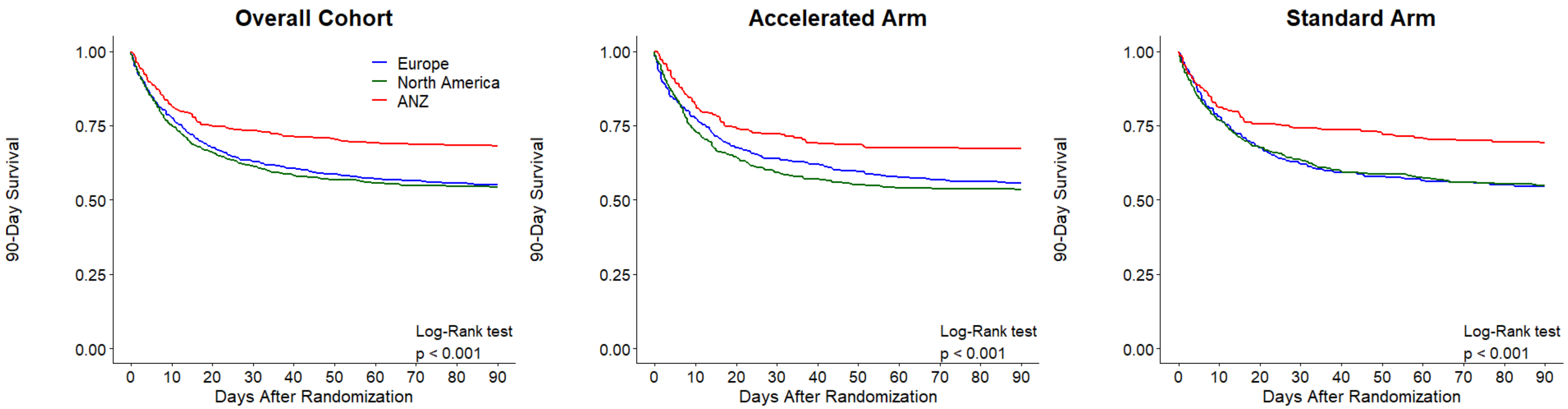

Overall Cohort – No. at Risk

	0	10	20	30	40	50	60	70	80	90
Europe	1143	888	773	719	692	670	652	644	637	629
North America	994	747	657	611	580	565	554	545	542	538
ANZ	556	454	417	407	397	392	385	382	380	379

Accelerated Arm – No. at Risk

	0	10	20	30	40	50	60	70	80	90
Europe	572	443	387	365	354	340	330	325	322	318
North America	497	365	320	295	283	274	268	267	267	266
ANZ	277	228	206	200	192	190	187	187	186	186

Standard Arm – No. at Risk

	0	10	20	30	40	50	60	70	80	90
Europe	571	445	386	354	338	330	322	319	315	311
North America	497	382	337	316	297	291	286	278	275	272
ANZ	279	226	211	207	205	202	198	195	194	193

eFigure 3 - Renal Replacement Therapy-Free Days According to Geographic Region

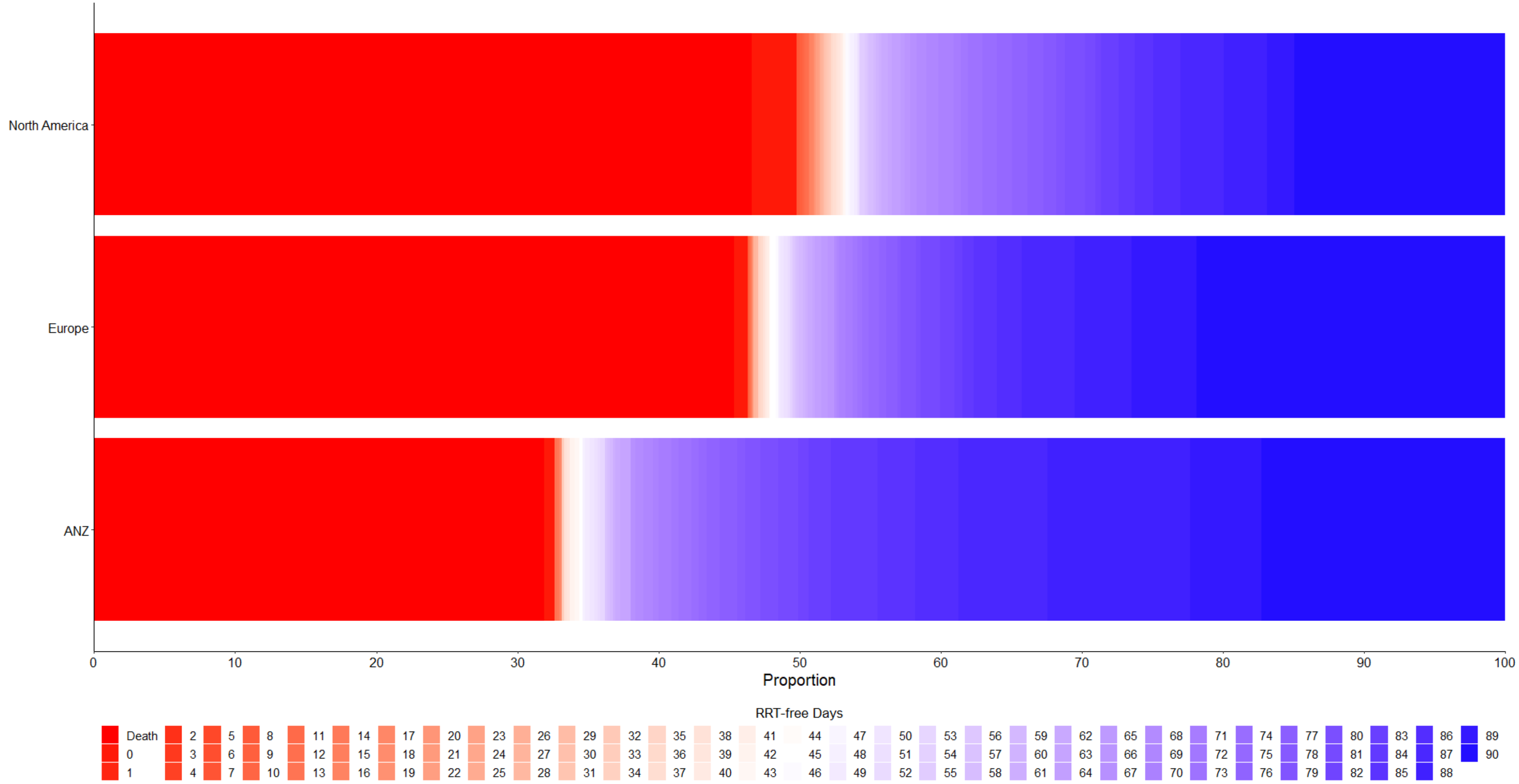

Renal replacement–free days as horizontally stacked proportions by geographic region. Red represents worse values and blue represents better values. The adjusted median difference from the primary analysis, using a median regression using an interior point algorithm, and are reported in Table 4.

STARRT-AKI Investigators

Institution	Name for Publication
**Steering Committee**	Sean M Bagshaw (Co-Chair); Ron Wald (Co-Chair); Neill K.J. Adhikari; Rinaldo Bellomo; Didier Dreyfuss; Bin Du; Martin P. Gallagher; Stéphane Gaudry; Eric A. Hoste; François Lamontagne; Michael Joannidis; Kathleen D. Liu; Daniel F. McAuley; Shay P. McGuinness; Alistair D. Nichol; Marlies Ostermann; Paul M. Palevsky; Haibo Qiu; Ville Pettilä; Antoine G. Schneider; Orla M. Smith; Suvi T. Vaara; Matthew Weir
**AUSTRALIA**	
Austin Health	Rinaldo Bellomo; Glenn M. Eastwood, Leah Peck; Helen Young
Princess Alexandra Hospital	Peter Kruger; Gordon Laurie; Emma Saylor; Jason Meyer; Ellen Venz; Krista Wetzig
Western Health	Craig French; Forbes McGain; John Mulder; Gerard Fennessy; Sathyajith Koottayi; Samantha Bates; Miriam Towns; Rebecca Morgan; Anna Tippett
The Alfred Hospital	Andrew Udy; Chris Mason; Elisa Licari; Dashiell Gantner; Jason McClure; Alistair Nichol; Phoebe McCracken; Jasmin Board; Emma Martin; Shirley Vallance; Meredith Young; Chelsey Vladic; Steve McGloughlin
Royal Prince Alfred Hospital	David Gattas; Heidi Buhr; Jennifer Coles; Debra Hutch; James Wun
Nepean Hospital	Louise Cole; Christina Whitehead; Julie Lowrey; Kristy Masters; Rebecca Gresham
Sunshine Coast University Hospital	Victoria Campbell; David Gutierrez; Jane Brailsford; Loretta Forbes; Lauren Murray; Teena Maguire
Barwon Health	Martina NiChonghaile; Neil Orford; Allison Bone; Tania Elderkin; Tania Salerno
Bendigo Health	Tim Chimunda; Jason Fletcher; Emma Broadfield; Sanjay Porwal; Cameron Knott; Catherine Boschert; Julie Smith
Ballarat Health	Angus Richardson; Dianne Hill
Eastern Health	Graeme Duke; Peter Oziemski; Santiago Cegarra; Peter Chan; Deborah Welsh; Stephanie Hunter; Owen Roodenburg; John Dyett; Nicos Kokotsis; Max Moser; Yang Yang; Laven Padayachee; Joseph Vetro; Himangsu Gangopadhyay; Melissa Kaufman
The Northern Hospital	Angaj Ghosh; Simone Said
Flinders Medical Centre	Alpesh Patel; Shailesh Bihari; Elisha Matheson; Xia Jin; Tapaswi Shrestha; Kate Schwartz
Concord Repatriation General Hospital	Martin P. Gallagher; Rosalba Cross; Winston Cheung; Helen Wong; Mark Kol; Asim Shah; Amanda Y. Wang
Prince of Wales Clinical School, University of New South Wales	Zoltan Endre
Royal North Shore Hospital	Celia Bradford; Pierre Janin; Simon Finfer; Naomi Diel; Jonathan Gatward; Naomi Hammond; Anthony Delaney; Frances Bass; Elizabeth Yarad
St. Vincent's Hospital	Hergen Buscher; Claire Reynolds; Nerilee Baker

**AUSTRIA**	
Department of Internal Medicine, Medical University Innsbruck	Michael Joannidis; Romuald Bellmann; Andreas Peer; Julia Hasslacher; Paul Koglberger; Sebastian Klein; Klemens Zotter; Anna Brandtner; Armin Finkenstedt; Adelheid Ditlbacher; Frank Hartig
Department of General and Surgical Critical Care Medicine, Medical University Innsbruck	Dietmar Fries; Mirjam Bachler; Bettina Schenk; Martin Wagner
Department of Internal Medicine, Medical University of Graz	Philipp Eller
Medical University of Vienna	Thomas Staudinger; Esther Tiller; Peter Schellongowski; Andja Bojic
**BELGIUM**	
Ghent University Hospital	Eric A. Hoste; Stephanie Bracke; Luc De Crop; Daisy Vermeiren
**BRAZIL**	
Hospital de Clínicas de Porto Alegre	Fernando Thome; Bianca Chiella; Lucia Fendt; Veronica Antunes
**CANADA**	
Centre de recherche de l'Hôpital Maisonneuve-Rosemont	Jean-Philippe Lafrance
Centre Hospitalier Universitaire de Sherbrooke	François Lamontagne; Frédérick D'Aragon; Charles St-Arnaud; Michael Mayette; Élaine Carbonnaeu; Joannie Marchand; Marie-Hélène Masse; Marilène Ladouceur
CHU de Québec-Université Laval Research Center	Alexis F. Turgeon; François Lauzier; David Bellemare; Charles Langis Francoeur; Guillaume LeBlanc; Gabrielle Guilbault; Stéphanie Grenier; Eve Cloutier; Annick Boivin; Charles Delisle-Thibault; Panagiota Giannakouros; Olivier Costerousse
Centre hospitalier de l'Université de Montréal	Jean-François Cailhier; François-Martin Carrier; Ali Ghamraoui; Martine Lebrasseur; Fatna Benettaib; Maya Salamé; Dounia Boumahni
Centre intégré universitaire de santé et de services sociaux de la Mauricie-et-du-Centre-du-Québec	Ying Tung Sia; Jean-François Naud; Isabelle Roy
Foothills Medical Centre	Henry T. Stelfox; Stacey Ruddell; Braden J. Manns
Grey Nuns Community Hospital	Shelley Duggan; Dominic Carney; Jennifer Barchard
Hamilton Health Sciences	Richard P. Whitlock; Emilie Belley-Cote; Nevena Savija; Alexandra Sabev; Troy Campbell; Thais Creary; Kelson Devereaux; Shira Brodutch
Health Sciences Centre, University of Manitoba	Claudio Rigatto; Bojan Paunovic; Owen Mooney; Anna Glybina; Oksana Harasemiw; Michelle Di Nella
Health Sciences North	John Harmon; Navdeep Mehta; Louis Lakatos; Nicole Haslam
Institut Universitaire de Cardiologie et de Pneumologie de Québec	Francois Lellouche; Mathieu Simon; Ying Tung; Patricia Lizotte; Pierre-Alexandre Bourchard
Juravinski Hospital	Bram Rochwerg; Tim Karachi; Tina Millen
Kingston Health Sciences Centre	John Muscedere; David Maslove; J. Gordon Boyd; Stephanie Sibley; John Drover; Miranda Hunt; Ilinca Georgescu

Lakeridge Health	Randy Wax; Ilan Lenga; Kavita Sridhar; Andrew Steele; Kelly Fusco; Taneera Ghate; Michael Tolibas; Holly Robinson
London Health Sciences Centre, University Hospital	Matthew A. Weir; Ravi Taneja
London Health Sciences Centre, Victoria Hospital	Ian M. Ball; Amit Garg; Eileen Campbell; Athena Ovsenek
Mazankowski Alberta Heart Institute	Sean M. Bagshaw, Sean van Diepen, Nadia Baig
McGill University Health Centre	Sheldon Magder; Han Yao; Ahsan Alam; Josie Campisi
Misericordia Community Hospital	Erika MacIntyre; Ella Rokosh; Kimberly Scherr
Mount Sinai Hospital	Stephen Lapinsky; Sangeeta Mehta; Sumesh Shah
Peter Lougheed Centre	Daniel J. Niven; Henry T. Stelfox; Stacey Ruddell
Red Deer Regional Hospital	Michael Russell; Kym Jim; Gillian Brown; Kerry Oxtoby; Adam Hall; Luc Benoit; Colleen Sokolowski
Regina Qu'Appelle Health Authority	Bhanu Prasad; Jag Rao; Shelley Giebel
Royal Alexandra Hospital	Demetrios J. Kutsogiannis; Patricia Thompson; Tayne Thompson
St. Joseph's Health Centre	Robert Cirone; Kanthi Kavikondala
St. Joseph's Healthcare	Mark Soth; France Clarke; Alyson Takaoka
St. Michael's Hospital	Ron Wald; David Mazer; Karen Burns; Jan Friedrich; David Klein; Gyan Sandhu; Marlene Santos; Imrana Khalid; Jennifer Hodder
St. Paul's Hospital	Peter Dodek; Najib Ayas; Victoria Alcuaz
Sturgeon Community Hospital	Gabriel Suen; Oleksa Rewa; Gurmeet Singh; Sean Norris; Neil Gibson; Castro Arias; Aysha Shami; Celine Pelletier
Sunnybrook Health Sciences Centre	Neill K.J. Adhikari; Alireza Zahirieh; Andre Amaral; Nicole Marinoff; Navjot Kaur; Adic Perez; Jane Wang
Surrey Memorial Hospital	Gregory Haljan; Christopher Condin
The Ottawa Hospital	Lauralyn McIntyre; Brigette Gomes; Rebecca Porteous; Irene Watpool; Swapnil Hiremath; Edward Clark
Toronto General Hospital	Margaret S. Herridge; Felicity Backhouse
Toronto Western Hospital	M. Elizabeth Wilcox; Karolina Walczak
Trillium Health Partners	Vincent Ki; Asheer Sharman; Martin Romano
University of Alberta Hospital	Sean M. Bagshaw; R.T. Noel Gibney; Adam S. Romanovsky; Oleksa Rewa; Lorena McCoshen; Nadia Baig
Vancouver Island Health Authority	Gordon Wood; Daniel Ovakim; Fiona Auld; Gayle Carney
**CHINA**	
Beijing Friendship Hospital, Capital Medical University	Meili Duan; Xiaojun Ji; Dongchen Guo; Zhili Qi; Jin Lin; Meng Zhang; Lei Dong; Jingfeng Liu; Pei Liu; Deyuan Zhi; Guoqiang

	Bai; Yu Qiu; Ziqi Yang; Jing Bai; Zhuang Liu; Haizhou Zhuang; Haiman Wang; Jian Li; Mengya Zhao; Xiao Zhou
Guizhou Provincial People's Hospital	Xianqing Shi; Baning Ye; Manli Liu; Jing Wu; Yongjian Fu; Dali Long; Yu Pan; Jinlong Wang; Huaxian Mei; Songsong Zhang; Mingxiang Wen; Enyu Yang; Sijie Mu; Jianquan Li; Tingting Hu
Henan Provincial People's Hospital	Bingyu Qin; Min Li; Cunzhen Wang; Xin Dong; Kaiwu Wang; Haibo Wang; Jianxu Yang
Peking Union Medical College Hospital	Bin Du; Chuanyao Wang
Peking University First Hospital	Dongxin Wang; Nan Li
Renmin Hospital of Wuhan University	Zhui Yu; Song Xu; Lan Yao; Guo Hou; Zhou Liu; Liping Lu; Yingtao Lian
Shandong Provincial Hospital	Chunting Wang; Jichen Zhang; Ruiqi Ding; Guoqing Qi; Qizhi Wang; Peng Wang; Zhaoli Meng; Man Chen; Xiaobo Hu
The First Affiliated Hospital of Bengbu Medical College	Xiandi He; Shibing Zhao; Lele Hang; Rui Li; Suhui Qin; Kun Lu; Shijuan Dun; Cheng Liu; Qi Zhou; Zhenzhen Chen; Jing Mei
The First Affiliated Hospital of Xiamen University	Minwei Zhang; Hao Xu; Jincan Lin
The First Affiliated Hospital of Xi'An Jiaotong University	Qindong Shi; Lijuan Fu; Qinjing Zeng; Hongye Ma; Jinqi Yan; Lan Gao; Hongjuan Liu; Lei Zhang; Hao Li; Xiaona He; Jingqun Fan; Litao Guo; Yu Liu; Xue Wang; Jingjing Sun
The First Hospital of Jilin University	Zhongmin Liu; Juan Yang; Lili Ding; Lulu Sheng; Xingang Liu
Wuxi People's Hospital	Jie Yan; Quihui Wang; Yifeng Wang; Dan Zhao
Xiangya Hospital Central South University	Shuangping Zhao; Chenghuan Hu; Jing Li; Fuxing Deng
Zhongda Hospital Southeast University	Haibo Qiu; Yi Yang; Min Mo; Chun Pan; Changde Wu; Yingzi Huang; Lili Huang; Airan Liu
**FINLAND**	
Helsinki University Hospital	Ville Pettilä; Suvi T. Vaara; Anna-Maija Korhonen; Sanna Törnblom; Sari Sutinen; Leena Pettilä; Jonna Heinonen; Eliria Lappi; Taria Suhonen
Tampere University Hospital	Sari Karlsson; Sanna Hoppu; Ville Jalkanen; Anne Kuitunen; Markus Levoranta; Jaakko Långsjö; Sanna Ristimäki; Kaisa Malila; Anna Wootten; Simo Varila
Turku University Hospital	Mikko J Järvisalo; Outi Inkinen; Satu Kentala; Keijo Leivo; Paivi Haltia
**FRANCE**	
Hôpital Louis Mourier	Didier Dreyfuss; Jean-Damien Ricard; Jonathan Messika; Abirami Tiagarajah; Malo Emery; Aline Dechanet; Coralie Gernez; Damien Roux
Centre Hospitalier Départemental La Roche-Sur-Yon	Laurent Martin-Lefevre; Maud Fiancette; Isabelle Vinatier; Jean Claude Lacherade; Gwenhaël Colin; Christine Lebert; Marie-Ange Azais; Aihem Yehia; Caroline Pouplet; Matthieu Henry- Lagarrigue; Amélie Seguin; Laura Crosby
Medical Intensive Care Unit, Amiens University Hospital	Julien Maizel; Dimitri Titeca-Beauport

Hôpital Pitie-Salpetriere	Alain Combes; Ania Nieszkowska; Paul Masi; Alexandre Demoule; Julien Mayaux; Martin Dres; Elise Morawiec; Maxens Decalvele; Suela Demiri; Morgane Faure; Clémence Marios; Maxime Mallet; Marie Amélie Ordon; Laura Morizot; Marie Cantien; François Pousset
Hôpital Avicenne/Hôpital Jean Verdier	Stéphane Gaudry; Florent Poirson; Yves Cohen
Hospices Civils de Lyon, Hôpital Edouard Herriot, Service de Médecine Intensive – Réanimation	Laurent Argaud; Martin Cour; Laurent Bitker; Marie Simon; Romain Hernu; Thomas Baudry; Sylvie De La Salle
CH De Bourg-en-Bresse – Fleyriat	Adrien Robine; Nicholas Sedillot; Xavier Tchenio; Camille Bouisse; Sylvie Roux
CHRU de Nîmes	Saber Davide Barbar; Rémi Trusson
Rouen University Hospital	Fabienne Tamion; Steven Grangé; Dorothée Carpentier
CH Sud Francilien	Guillaume Chevrel; Luis Ensenyat-Martin; Sophie Marque
CHU Dijon	Jean-Pierre Quenot; Pascal Andreu; Auguste Dargent; Audrey Large
CH Le Mans - Réanimation Medico - Chirurgicale	Nicolas Chudeau; Mickael Landais; Benoit Derrien; Jean Christophe Callahan; Christophe Guitton; Charlène Le Moal; Alain Robert
CHU Nantes/Service d'Anesthésie - Réanimation chirurgicale HD PTMC	Karim Asehnoune; Raphaël Cinotti; Nicolas Grillot; Dominique Demeure
Germon et Gauthier Hospital – Béthune	Christophe Vinsonneau; Imen Rahmani; Mehdi Marzouk; Thibault Dekeyser; Caroline Sejourne; Mélanie Verlay; Fabienne Thevenin; Lucie Delecolle
Centre Hospitalier Lens	Didier Thevenin
Clermont Ferrand	Bertrand Souweine; Elisabeth Coupez; Mireille Adda
CH de Dieppe	Jean-Pierre Eraldi; Antoine Marchalot
Hôpital Henri Mondor	Nicolas De Prost; Armand Mekontso Dessap; Keyvan Razazi
Hôpital Civil	Ferhat Meziani; Julie Boisrame-Helms; Raphael Clere-Jehl; Xavier Delabranche; Christine Kummerlen; Hamid Merdji; Alexandra Monnier; Yannick Rabouel; Hassene Rahmani; Hayat Allam; Samir Chenaf; Vincenta Franja
CHU de Pointe à Pitre	Bertrand Pons; Michel Carles; Frédéric Martino; Régine Richard
André Mignot	Benjamin Zuber; Guillaume Lacave
CHU de Nantes	Karim Lakhal; Bertrand Rozec; Hoa Dang Van
Centre de Beaumont sur Oise	Éric Boulet
Centre Hospitalier René Dubos Pontoise	Fouad Fadel; Cedric Cleophax; Nicolas Dufour; Caroline Grant; Marie Thuong
Hotel Dieu – Service de Médicale	Jean Reignier; Emmanuel Canet; Laurent Nicolet
CHR Orleans	Thierry Boulain; Mai-Anh Nay; Dalila Benzekri; François Barbier; Anne Bretagnol; Toufik Kamel; Armelle Mathonnet; Grégoire Muller; Marie Skarzynski; Julie Rossi; Amandine Pradet; Sandra Dos Santos; Aurore Guery; Lucie Muller; Luis Felix
CH Lyon Sud – Pierre Benite	Julien Bohé; Guillaume Thiéry

Universite de Paris, Hopital Europeen Georges Pompidou	Nadia Aissaoui; Damien Vimpere; Morgane Commeureuc; Jean-Luc Diehl; Emmanuel Guerot
**GERMANY**	
Klinikum Coburg	Orfeas Liangos; Monika Wittig
University Hospital Münster	Alexander Zarbock; Mira Küllmar; Thomas van Waegeningh; Nadine Rosenow
**IRELAND**	
St. Vincent's University Hospital	Alistair D. Nichol; Kathy Brickell; Peter Doran; Patrick T. Murray
**ITALY**	
IRCCS San Raffaele Scientific Institute	Giovanni Landoni; Rosalba Lembo; Alberto Zangrillo; Giacomo Monti; Margherita Tozzi; Matteo Marzaroli; Gaetano Lombardi
San Carlo Hospital	Gianluca Paternoster; Michelangelo Vitiello
**NEW ZEALAND**	
Cardiovascular Surgical Intensive Care Unit, Auckland Hospital	Shay McGuinness; Rachael Parke; Magdalena Butler; Eileen Gilder; Keri-Anne Cowdrey; Samantha Wallace; Jane Hallion; Melissa Woolett; Philippa Neal; Karina Duffy;<br>Stephanie Long
Department of Critical Care Medicine, Auckland Hospital	Colin McArthur; Catherine Simmonds; Yan Chen; Rachael McConnochie; Lynette Newby
Christchurch Hospital	David Knight; Seton Henderson; Jan Mehrtens; Stacey Morgan; Anna Morris; Kymbalee Vander Hayden; Tara Burke
Hawke's Bay Hospital	Matthew Bailey; Ross Freebairn; Lesley Chadwick; Penelope Park; Christine Rolls; Liz Thomas
Rotorua Hospital	Ulrike Buehner; Erin Williams
Taranaki Hospital	Jonathan Albrett; Simon Kirkham; Carolyn Jackson
Tauranga Hospital	Troy Browne; Jennifer Goodson; David Jackson; James Houghton; Owen Callender; Vicki Higson; Owen Keet; Clive Dominy
Wellington Hospital	Paul Young; Anna Hunt; Harriet Judd; Cassie Lawrence; Shaanti Olatunji; Yvonne Robertson; Charlotte Latimer-Bell; Deborah Hendry; Agnes Mckay-Vucago; Nina Beehre; Eden<br>Lesona; Leanlove Navarra; Chelsea Robinson
Whangarei Hospital	Ryan Jang; Andrea Junge; Bridget Lambert
**SWITZERLAND**	
Centre Hospitalier Universitaire Vaudois	Antoine G. Schneider; Michel Thibault; Philippe Eckert; Sébastien Kissling; Erietta Polychronopoulos; Elettra Poli; Marco Altarelli; Madeleine Schnorf; Samia Abed Mallaird
Hôpitaux Universitaires de Genève	Claudia Heidegger; Aurelie Perret; Philippe Montillier; Frederic Sangla; Seigenthaller Neils; Aude De Watteville
**UNITED KINGDOM**	
Barking, Havering and Redbridge University Hospitals NHS Trust	Mandeep-Kaur Phull; Aparna George; Nauman Hussain; Tatiana Pogreban

Barnsley Hospital NHS Foundation Trust	Steve Lobaz; Alison Daniels; Mishell Cunningham; Deborah Kerr; Alice Nicholson
Buckinghamshire Healthcare NHS Trust	Pradeep Shanmugasundaram; Judith Abrams; Katarina Manso; Geraldine Hambrook; Elizabeth McKerrow; Juvy Salva; Stephen Foulkes
Cardiff and Vale University Health Board	Matthew Wise; Matt Morgan; Jenny Brooks; Jade Cole; Tracy Michelle Davies; Helen Hill; Emma Thomas
Chelsea and Westminster Hospital NHS Foundation Trust	Marcela Vizcaychipi; Behrad Baharlo; Jaime Carungcong; Patricia Costa; Laura Martins
East Kent NHS Trust	Ritoo Kapoor; Tracy Hazelton; Angela Moon; Janine Musselwhite
Golden Jubilee National Hospital, NHS Scotland	Ben Shelley; Philip McCall
Guy's and St. Thomas' NHS Foundation Trust	Marlies Ostermann; Gill Arbane; Aneta Bociek; Martina Marotti; Rosario Lim; Sara Campos; Neus Grau Novellas; Armando Cennamo; Andrew Slack; Duncan Wyncoll; Luigi Camporota; Simon Sparkes; Rosalinde Tilley
University Hairmyres Hospital, NHS Lanarkshire	Austin Rattray; Gayle Moreland; Jane Duffy; Elizabeth McGonigal
King's College Hospital NHS Foundation Trust	Philip Hopkins; Clare Finney; John Smith; Harriet Noble; Hayley Watson; Claire-Louise Harris; Emma Clarey; Eleanor Corcoran
Leeds Teaching Hospital NHS Foundation Trust	James Beck; Clare Howcroft; Nora Youngs; Elizabeth Wilby; Bethan Ogg
Lincoln County Hospital – United Lincolnshire Hospitals NHS Foundation Trust	Adam Wolverson; Sandra Lee; Susie Butler; Maryanne Okubanjo; Julia Hindle
Liverpool University Hospitals NHS Foundation Trust	Ingeborg Welters; Karen Williams; Emily Johnson; Julie Patrick-Heselton; David Shaw; Victoria Waugh
Milton Keynes University Hospital NHS Foundation Trust	Richard Stewart; Esther Mwaura; Lynn Wren; Louise Mew; Sara-Beth Sutherland; Jane Adderley
University Hospital Monklands, NHS Lanarkshire	Jim Ruddy; Margaret Harkins
NHS Grampian	Callum Kaye; Teresa Scott; Wendy Mitchell; Felicity Anderson; Fiona Willox
North Tees and Hartlepool Foundation NHS Trust	Vijay Jagannathan; Michele Clark; Sarah Purv
Nottingham University Hospital - Queen's Medical Centre	Andrew Sharman; Megan Meredith; Lucy Ryan; Louise Conner; Cecilia Peters; Dan Harvey
Queen Elizabeth Hospital - Lewisham and Greenwich NHS Trust	Ashraf Roshdy; Amy Collins
Queen Elizabeth University Hospital	Malcolm Sim; Steven Henderson
Royal Bournemouth & Christchurch Hospitals NHS Trust	Nigel Chee; Sally Pitts; Katie Bowman; Maria Dilawershah; Luke Vamplew; Elizabeth Howe

Royal Brompton and Harefield NHS Foundation Trust	Paula Rogers; Clara Hernandez; Clara Prendergast; Jane Benton; Alex Rosenberg
Royal Surrey County Hospital NHS Foundation Trust	Lui G. Forni; Alice Grant; Paula Carvelli
Sheffield Teaching Hospitals NHS Foundation Trust	Ajay Raithatha; Sarah Bird; Max Richardson; Matthew Needham; Claire Hirst
St. George's University Hospitals NHS Foundation Trust	Jonathan Ball; Susannah Leaver; Luisa Howlett; Carlos Castro Delgado; Sarah Farnell-Ward; Helen Farrah; Geraldine Gray; Gipsy Joseph; Francesca Robinson
St. Helen's and Knowsley Teaching Hospitals NHS Trust	Ascanio Tridente; Clare Harrop; Karen Shuker
University Hospital Ayr, NHS Ayrshire & Arran	Derek McLaughlan; Judith Ramsey; Sharon Meehan
University Hospital Lewisham, Lewisham and Greenwich NHS Trust	Bernd Oliver Rose; Rosie Reece-Anthony; Babita Gurung
University Hospitals Birmingham NHS Foundation Trust	Tony Whitehouse; Catherine Snelson; Tonny Veenith; Andy Johnston; Lauren Cooper; Ron Carrera; Karen Ellis; Emma Fellows; Samanth Harkett; Colin Bergin; Elaine Spruce; Liesl Despy; Stephanie Goundry; Natalie Dooley; Tracy Mason; Amy Clark
University Hospitals Coventry and Warwickshire NHS Trust	Gemma Dignam; Geraldine Ward
Warwick Hospital, South Warwickshire NHS Trust	Ben Attwood; Penny Parsons; Sophie Mason
St. Richard's Hospital, Western Sussex Hospitals NHS Foundation Trust	Michael Margarson; Jenny Lord; Philip McGlone
Worthing Hospital, Western Sussex Hospitals NHS Foundation Trust	Luke E. Hodgson; Indra Chadbourn; Raquel Gomez; Jordi Margalef
York Teaching Hospital NHS Foundation Trust	Rinus Pretorius; Alexandra Hamshere; Joseph Carter; Hazel Cahill; Lia Grainger; Kate Howard; Greg Forshaw; Zoe Guy
**UNITED STATES**	
Mayo Clinic, Rochester	Kianoush B. Kashani; Robert C. Albright Jr.; Amy Amsbaugh; Anita Stoltenberg; Alexander S. Niven
Rhode Island Hospital	Matthew Lynch; AnnMarie O'Mara; Syed Naeem; Sairah Sharif; Joyce McKenney Goulart
The Miriam Hospital	Matthew Lynch; AnnMarie O'Mara; Syed Naeem; Sairah Sharif; Joyce McKenney Goulart
University of Alabama at Birmingham	Ashita Tolwani; Claretha Lyas; Laura Latta
University of Florida	Azra Bihorac; Haleh Hashemighouchani; Philip Efron; Matthew Ruppert; Julie Cupka; Sean Kiley; Joshua Carson; Peggy White; George Omalay; Sherry Brown; Laura Velez; Alina Marceron
University of Kentucky	Javier A. Neyra; Juan Carlos Aycinena; Madona Elias; Victor

	M. Ortiz-Soriano; Caroline Hauschild; Robert Dorfman
